# Psychological Stress Associated Bile Acid Reprogramming Promotes Hepatocellular Carcinoma Progression

**DOI:** 10.1002/advs.76416

**Published:** 2026-07-11

**Authors:** Ruijiang Zeng, Mengmeng Wang, Kang Wang, Zhuo Xing, Yulong Hong, Tongtong Li, Geng Zong, Chong Yang, Dan Zhang, Zhangling Chen, Xin Jin

**Affiliations:** ^1^ Department of Urology The Second Xiangya Hospital Central South University Changsha Hunan China; ^2^ Key Laboratory of Diabetes Immunology (Central South University), Ministry of Education National Clinical Research Center for Metabolic Disease Changsha China; ^3^ Uro‐Oncology Institute of Central South University Changsha Hunan China; ^4^ Hunan Key Laboratory of Tumor Models and Individualized Medicine The Second Xiangya Hospital Central South University Changsha Hunan China; ^5^ Cancer Center, Union Hospital, Tongji Medical College Huazhong University of Science and Technology Wuhan China; ^6^ Department of Cardiology The First Affiliated Hospital of University of South China Hengyang P. R. China; ^7^ CAS Key Laboratory of Nutrition, Metabolism and Food Safety, Shanghai Institute of Nutrition and Health, University of Chinese Academy of Sciences Chinese Academy of Sciences Shanghai China; ^8^ Department of Hepatobiliary and Pancreas Surgery, Sichuan Provincial People's Hospital School of Medicine, University of Electronic Science and Technology of China Chengdu Sichuan China; ^9^ Department of Cardiovascular Medicine The Second Xiangya Hospital of Central South University Changsha P. R. China; ^10^ FuRong Laboratory Changsha Hunan China; ^11^ Key Laboratory of Cardiometabolic Medicine in Hunan Province China

**Keywords:** CBX5, ferroptosis, psychological stress, social isolation, taurocholate

## Abstract

Psychological stress, particularly depression, is increasingly recognized as a non‐genetic determinant of cancer progression, yet its role in hepatocellular carcinoma (HCC) remains unclear. By integrating three prospective cohorts (CHARLS, NHANES, and UK Biobank; n = 492,501), we show that depression was associated with increased HCC risk (CHARLS: hazard ratio (HR) = 2.28, 95% confidence interval (CI) 1.06–4.93; NHANES: odds ratio (OR) = 5.95, 95% CI 2.42–14.0; UK Biobank: HR = 1.39, 95% CI 1.10–1.79). Using patient samples, multi‐omics analyses, and social isolation (SI) models, we identified taurocholate as a key metabolite elevated in psychological stress‐associated HCC. Taurocholate stabilizes CBX5 by weakening its interaction with E3 ubiquitin ligases. CBX5 cooperated with MYC to activate PHGDH transcription, thereby reducing ferroptotic sensitivity and promoting tumor growth. Importantly, ursodeoxycholic acid (UDCA), a clinically approved bile acid modulator, reduced taurocholate levels and suppressed tumor growth in SI‐associated HCC models. Together, these findings support a mechanistic link between depression and HCC progression via bile acid metabolic reprogramming and identify the taurocholate‐CBX5‐MYC‐PHGDH axis as a potential therapeutic target.

## Graphical abstract

Depression is increasingly recognized as a risk factor for chronic diseases, yet its biological impact on cancer remains unclear. Using data from more than 490 000 participants across three international cohorts, we show that depression significantly increases the risk of liver cancer. Integrating these population findings with patient and animal studies, we identify the bile acid taurocholate as a key link between depression and liver cancer progression. Elevated taurocholate promotes tumor growth, while the clinically approved bile acid drug ursodeoxycholic acid can reverse this effect. These findings reveal a direct biological connection between emotional stress and cancer, providing new insight for prevention and treatment strategies.

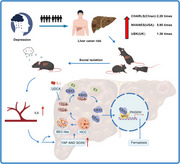

## 1 Introduction

Psychological stress, particularly depression, has emerged as an important regulator of systemic physiology and disease progression [1, 2, 3]. Increasing evidence suggests that chronic emotional disturbance can influence tumor biology through neuroendocrine‐immune pathways, yet its impact on hepatocellular carcinoma (HCC) remains poorly defined [4, 5, 6, 7, 8, 9, 10].

Psychiatric disorders and cancer are major contributors to the global disease burden [11, 12]. Depression has been associated with adverse cancer outcomes, including advanced‐stage diagnosis and reduced overall survival [13, 14, 15, 16]. These observations raise the possibility that depression may actively shape tumor progression rather than merely representing a comorbidity. However, the mechanisms underlying this association remain unclear.

The COVID‐19 pandemic further intensified this problem. Lockdown measures and social isolation (SI) increased the prevalence of depression and anxiety worldwide [17]. During the pandemic, survival outcomes for patients declined [18], and several studies linked SI and depression to poorer quality of life in HCC patients [19, 20]. A systematic review involving 64 247 participants reported that over one‐fifth of patients exhibit symptoms, with prevalence reaching 66.67% in some Southeast Asian populations [21]. Nevertheless, prospective evidence remains limited, and the molecular pathways by which depression on HCC progression have not been fully resolved.

To address these gaps, we integrated data from three large prospective cohorts: China Health and Retirement Longitudinal Study (CHARLS), National Health and Nutrition Examination Survey (NHANES), and UK Biobank (UKB) with mechanistic studies using experimental models. This approach identified taurocholate as a key metabolite linking depression to HCC progression. Mechanistically, taurocholate regulated ferroptosis. We further explored the potential of bile acid modulation with ursodeoxycholic acid (UDCA). These findings provide a mechanistic framework linking psychological stress, bile acid reprogramming, and HCC progression.

## 2 Materials and Methods

### 2.1 Study Population and Design

Our population‐based analyses were conducted based on the CHARLS [22], NHANES [23] and UKB [24]. The CHARLS is a prospective cohort study of middle‐aged and older Chinese people, which collected a set of high‐quality microdata representing households and individuals across China to analyze the problem of population ageing, promote interdisciplinary research on ageing, and provide a scientific basis for the development and improvement of relevant policies [22]. The baseline survey was conducted in 2011 using multi‐stage probability sampling, with participants covering more than 10 000 households in 28 provinces, 150 counties and 450 villages in China. Follow‐up surveys were conducted in 2013, 2015, and 2018 [22]. In this current analysis, 18 245 middle‐age and older participants from baseline were initially included. We further excluded participants who had liver cancer at baseline, missing data on depression score, loss of follow‐up. Finally, 14 770 individuals from the CHARLS were included in the analysis (Figure S1).

The NHANES aims to survey the health and nutritional status of the U.S. population [23], which includes a random sample of 15 counties nationwide, with every 2 years serving as a cycle. Its uniqueness lies in the combination of structured questionnaire interviews with complete physical examinations. Interviews were conducted within respondents' homes, while physical measurements were taken at specialized and well‐equipped mobile centers [23]. For the current analysis, 57 381 participants were initially enrolled from 6 survey cycles of NHANES (2007–2008, 2009–2010, 2011–2012, 2013–2014, 2015–2016 and 2017–2018). We further excluded individuals aged <20 years, or those with missing data on liver cancer, or depression score. Finally, 29 983 participants from the NHANES were included in the analysis (Figure S1).

The UKB is a large prospective cohort study that recruited approximately 500 000 UK residents aged 40–69 during 2006 to 2010 (baseline), which recorded a variety of information on participants' demographic, lifestyle, laboratory test, genome, and imaging examination, aiming to promote the study of human health and disease at baseline and follow‐up [24]. We initially included 501 209 participants. Of which, we further excluded participants who did not have depression score, or who had diagnosis of liver cancer at baseline. Finally, 447 748 individuals from the UKB cohort were analyzed (Figure S1).

### 2.2 Ethics Statement 

The CHARLS study was ethically reviewed and approved by the Biomedical Ethics Review Committee of Peking University (IRB00001052‐11015). The NHANES study protocol was approved by the Research Ethics Review Board of the National Center for Health Statistics (NCHS), and the UKB study was approved by the North West Multi‐centre Research Ethics Committee and operates under the UK Biobank Ethics and Governance Framework and a Human Tissue Authority license. All participants in the three cohort studies signed informed consent.

### 2.3 Assessment of Depression

The ten‐item center for epidemiologic studies depression scale (CESD‐10) was used to screen depressive symptoms in the CHARLS study. The reliability and validity of CESD‐10 has been well established [25, 26]. Respondents were asked to answer 10 questions related to depressive symptoms, such as “Have you felt lonely in the past week?” and “Have you had trouble sleeping in the past week?.” The different frequencies of symptoms corresponded to a score of 0–3 (score range: 0–30). Depression degrees were defined according to the CESD‐10, with ≥10 being moderate to severe, 5–9 defined as mild, and ≤4 as no [27].

In the NHANES study, the nine‐item Patient Health Questionnaire (PHQ‐9) depression scale was employed to assess the frequency of symptoms experienced and reported by participants in the past 2 weeks, for example lack of interest in doing things, appetite or overeating, etc. The frequency of each symptom corresponded to a score between 0 and 3. The PHQ‐9 score scale ranges from 0–27, with ≤4, 5–9, and ≥10 identified as no, mild, and moderate to severe depressive symptoms, respectively [28]. The PHQ‐9 has been widely validated for diagnosing depression and its degree, with scores of 10 or higher having a sensitivity of 88% and specificity of 88% for diagnosing severe depression [29, 30].

We used the four‐item Patient Health Questionnaire (PHQ‐4) to assess depression in the UKB study. It was developed as a composite four‐item scale combining the Patient Health Questionnaire‐2 (PHQ‐2) and the 2‐item Generalized Anxiety Disorder Scale (GAD‐2) [31]. Specifically, respondents were asked respectively how often they had been bothered by the following four problems in the past 2 weeks, including depressed mood, disinterest, tenseness, and tiredness, with different frequencies corresponding to a score of 0–3. The total score ranged from 0–12, with ≥6 being diagnosed as depression [31]. The validity of the PHQ‐4 has been extensively tested and demonstrated to be associated with relevant self‐reported scales and demographic risk factors for depression in the general population [31, 32, 33].

### 2.4 Identification of Outcome

The primary outcome of this study was the incidence of liver cancer. In CHARLS and NHANES, liver cancer was identified based on self‐reported physician diagnosis. Specifically, participants were asked ‘Have you been told by your doctor which chronic disease you have?’. Participants who answered, ‘cancer or malignant tumor’ were then asked ‘which organ or part has cancer?’, participants who reported ‘liver’ were defined as liver cancer. Participants who answered “no” or reported other diseases were not classified as having liver cancer. At the UKB, we were electronically connected to hospitalization and cancer registers to get information on liver cancer, and liver cancer was defined as C22 based on the 10th version of the International Classification of Diseases (ICD‐10). Participants were followed from baseline assessment until the date of liver cancer diagnosis, death, loss to follow‐up, or the end of follow‐up, whichever occurred first. The end of follow‐up was 31 August 2018 for CHARLS and 31 October 2022 for the UK Biobank.

In addition, lung, stomach, thyroid, colorectal, prostatic, breast, and cervical cancers were also identified by self‐reported questionnaires in CHARLS and NHANES. In the UKB, the ICD‐10 codes for these cancers were C34, C16, C73, C18‐C20, C61, C50, and C53, respectively.

### 2.5 Covariates

Covariates included age, sex, race and ethnicity (NHANES and UKB only), education level, marital status, economic status, physical activity, alcohol drinking status, smoking status, healthy eating index (HEI, NHANES and UKB only), body mass index (BMI), and history of chronic diseases. These covariates were derived from baseline assessments within each cohort, including questionnaires, physical examinations, and/or laboratory measurements where available.

Notably, the living standard was used to assess economic status and was classified as poor, low, average and high in CHARLS study. Income poverty ratio, as a continuous variable, was used to evaluate the economic situation in NHANES study. The Townsend Deprivation Index (TDI) was used to quantify the economic level of participants in UKB. History of chronic diseases, including hepatitis B virus (HBV) and hepatitis C virus (HCV) infection, was ascertained via self‐reported physician diagnosis in CHARLS and UKB, and via laboratory testing in NHANES. The HEI was computed by summing scores for 13 vital dietary components, reflecting compliance to the 2015–2020 Dietary Guidelines for Americans in NHANES and UKB [34]. A higher HEI score reflects a higher diet quality.

### 2.6 Cell Culture

Human hepatocellular carcinoma cell lines, Huh7 and Hep3B, were obtained from the Cell Bank of the Chinese Academy of Sciences (Shanghai, China). Huh7 cells were cultured in complete Dulbecco's Modified Eagle Medium (DMEM), and Hep3B cells were cultured in Minimum Essential Medium (MEM), both supplemented with 10% fetal bovine serum (FBS) and 1% penicillin‐streptomycin. The cells were maintained at 37°C in a humidified incubator with 5% CO_2_. Prior to use, all cell lines were authenticated by short tandem repeat (STR) DNA profiling and tested for mycoplasma contamination using the Universal Mycoplasma Detection Kit (ATCC 30–1012K); no mycoplasma contamination was detected.

### 2.7 Patient Specimens

In this study, we obtained 60 hepatocellular carcinoma tissue specimens and 100 plasma samples from patients whose diagnoses were all confirmed histologically. Sample collection was approved by the Ethics Committee of Sichuan Provincial People's Hospital, University of Electronic Science and Technology of China (Approval No. 2025573), and written informed consent was obtained from all participants. Fresh tissues were processed immediately after surgical removal; portions used for molecular assays were snap‐frozen in liquid nitrogen and stored at −80°C, whereas tissues used for histological assays were fixed and embedded according to standard pathology procedures. Peripheral blood was collected in anticoagulant tubes before treatment when possible, centrifuged to separate plasma, aliquoted to avoid repeated freeze‐thaw cycles, and stored at −80°C. Each patient completed psychometric assessments using the PHQ‐9 scales. Patients with scores of PHQ‐9 ≥5 were used to define clinically relevant emotional distress. The collected data are summarized in Table S5.

### 2.8 Chemicals and Reagents

DMEM, MEM, and 10% fetal bovine serum (FBS) were purchased from Gibco Biotechnology Co. Ltd. RIPA Lysis Buffer (#P0013) and BCA Protein Assay Kit (#P0010) were obtained from Beyotime Biotechnology (China). Lipofectamine 2000 (#11668030) was purchased from Thermo Fisher Scientific. Opti‐MEM (#31985070) was also acquired from Gibco Biotechnology Co. Ltd. Puromycin (#HY‐K1057) was obtained from MedChemExpress (Shanghai, China). Erastin (#S7242) and sodium taurocholate (#E0154) were purchased from Selleck. Taurocholate(#HY‐N0545) was purchased from MedChemExpress (MCE). Biotin‐Taurocholate(#R‐KKL45) was purchased from Xi'an ruixi Biological Technology Co.,Ltd.

### 2.9 Co‐Immunoprecipitation (Co‐IP) and Western Blot

Tissues and cells were lysed on ice using RIPA lysis buffer (Beyotime P0013B) supplemented with 1% protease inhibitor cocktail (GLPBIO GK10019). The lysates were gently rotated at 4°C for 30 min and then centrifuged at 12 100 rpm for 15 min at 4°C. Equal amounts of clarified lysate were incubated overnight at 4°C with the indicated primary antibody or species‐matched IgG control, followed by incubation with Protein A/G agarose beads (Thermo Fisher Scientific, USA). The beads were washed five times with lysis buffer on ice and then subjected to Western blot analysis according to standard protocols. The antibodies used are listed in Table S6.

### 2.10 RNA Extraction and Quantitative RT‐PCR

Total RNA was extracted using TRIzol reagent (Thermo Fisher Scientific, USA), assessed for concentration and purity, and reverse‐transcribed into cDNA using the PrimeScript RT Kit (#RR037A). Real‐time PCR amplification and detection were performed using TB Green Fast qPCR Mix (#RR430A) according to the manufacturer's instructions. β‐Actin was used as the internal reference gene. Relative expression was calculated using the 2−ΔΔCt method. The primer sequences are provided in Table S7.

### 2.11 RNA Interference and Overexpression

Small interfering RNAs (siRNAs) were purchased from GenePharma. Transfections were performed according to the manufacturer's instructions. Briefly, siRNA and Lipofectamine 2000 reagent were each diluted in serum‐free medium, combined, and incubated for 30 min at room temperature to form complexes. These complexes were then added to the cells, which were harvested 48 h post‐transfection for knockdown validation and downstream assays. The sequences of the siRNAs are listed in Table S8. Plasmids were obtained from GeneChem (Shanghai, China). Huh7 and Hep3B cells cultured in 10 cm^2^ dishes were transfected with 10 µg of plasmid DNA and 10 µL of Lipofectamine 2000 per dish, following the manufacturer's protocol. Transfection was carried out for 48 h to overexpress specific genes.

### 2.12 Chromatin Immunoprecipitation (ChIP) and ChIP‐qPCR

ChIP was performed using the Chromatin Extraction Kit and the ChIP Kit Magnetic—One Step (both from Abcam, USA). The purified DNA was then analyzed by quantitative PCR (ChIP‐qPCR). Enrichment at target loci was normalized to input DNA and compared with IgG controls. The primer sequences used are provided in Table S9.

### 2.13 Cell Counting Kit‐8 (CCK‐8)

Cells subjected to different treatments were seeded into 96‐well plates at a density of 1000 cells per well in 200 µL of culture medium. At designated time points, the medium was removed and replaced with 100 µL of fresh medium containing 10 µL of Cell Counting Kit‐8 (CCK‐8) reagent (Beyotime, China). After incubation, the absorbance at 450 nm was measured for each well using a microplate reader (Thermo Fisher Scientific, USA). Blank wells containing medium and CCK‐8 reagent but no cells were used for background correction.

### 2.14 Colony Formation Assay

Cells from different treatment groups were seeded into 6‐well plates at a density of 1000 cells per well and cultured in medium containing 10% fetal bovine serum (FBS) for approximately 8 to 14 days to allow colony formation. After the incubation period, the medium was removed, and the cells were gently rinsed twice with phosphate‐buffered saline (PBS). The cells were then fixed with 4% paraformaldehyde at room temperature for 30 min, followed by staining with crystal violet for 30 min. Excess stain was removed by rinsing with water, and the plates were air‐dried. Colonies containing more than 50 cells were photographed and counted.

### 2.15 Reactive Oxygen Species (ROS) Measurement

Cellular ROS levels were measured using the fluorescent probe DCFH‐DA (Beyotime). Huh7 and Hep3B cells cultured in 6‐well plates were incubated with 1 mL of serum‐free medium containing 5 µM DCFH‐DA at 37°C for 30 min in the dark. Subsequently, the cells were washed three times with serum‐free medium to remove excess probe. The mean fluorescence intensity of DCF was measured by flow cytometry. Additionally, fluorescence signals were quantified using fluorescence microscopy and analyzed with ImageJ software. Microscopy images were acquired under identical exposure settings.

### 2.16 Measurement of Malondialdehyde (MDA) and Glutathione (GSH) Levels

Cells from the different treatment groups were digested with 0.25% trypsin and washed twice with phosphate‐buffered saline (PBS). The cells were then lysed by ultrasonication on ice. The cell homogenate was centrifuged at 13 000 ± g for 15 min at 4°C to obtain the supernatant. Levels of MDA and GSH in the supernatant were measured using the MDA Assay Kit (#S0131S) and GSH Assay Kit (#S0053) from Beyotime Biotechnology Institute, respectively, according to the manufacturer's instructions. Results were normalized to total protein concentration to account for differences in cell number.

### 2.17 Transmission Electron Microscopy (TEM)

Cells (1 × 10^7^ per group) were collected and washed three times with phosphate‐buffered saline (PBS). Cells were fixed in 2.5% glutaraldehyde at 4°C. Following centrifugation, cell pellets were embedded in agarose and post‐fixed with 1% osmium tetroxide (OsO_4_; Ted Pella Inc., USA). Subsequently, the samples underwent graded ethanol dehydration at room temperature using increasing concentrations of ethanol. The resin‐infiltrated and embedded samples were polymerized in an oven at 65°C and then sectioned into ultrathin slices using an ultramicrotome (Leica, Germany). Finally, the cells were observed and images were captured using a transmission electron microscope (HT7800/HT7700, Hitachi, Japan).

### 2.18 Social Isolation Protocol

Male C57BL/6 mice were randomly assigned to either SI or GH conditions starting at 6 weeks of age. In the SI group, mice were housed individually in standard cages, while GH mice were housed in groups of 2–3. All animals had ad libitum access to food and water. Behavioral testing was conducted at the end of the 4‐week housing period. This timing was chosen to remain consistent with the tumor‐model schedule unless a specific experiment stated otherwise. All animal procedures were approved by the Ethics Committee of the Shouzheng Pharma (Wuhan) Biotechnology Co., Ltd. (Approval No. 2024112601).

Cbx5 heterozygous mice (Cbx5^+/–^) on a C57BL/6 background were initially generated by the Shanghai Model Organisms Center (Shanghai, China). These mice were then transferred to Schulerbauer Biotechnology Co. Ltd (Wuhan, China) for colony expansion and experimental breeding. Wild‐type (Cbx5^+/+^) and knockout (Cbx5–^/–^) littermates, obtained by intercrossing heterozygous pairs (Cbx5^+/–^ × Cbx5^+/–^), were used for subsequent experiments.

### 2.19 Behavioral Testing

To minimize acute stress responses and ensure the reliability of behavioral data, all mice were acclimated to the behavioral testing room for at least 30 min prior to the onset of each test. Behavioral assays were conducted in the order of increasing stress intensity to avoid potential carry‐over effects from more stressful procedures. Specifically, the open field test (OFT) was conducted on day 1, followed by the tail suspension test (TST) on day 2, and the forced swimming test (FST) on day 3. Each test was performed at the same time of day with 24‐h intervals between sessions to minimize cumulative stress and circadian variability. Apparatuses were cleaned between animals, and behavioral videos were analyzed using the same criteria across groups.

### 2.20 Open Field Test

Mice were individually placed in a 50 × 50 × 40 cm open‐field arena and allowed to explore freely for 5 min while being video‐recorded. The total distance traveled was quantified to assess general locomotor activity, and the time spent in the central versus peripheral zones was analyzed as an index of anxiety‐like behavior.

### 2.21 Tail Suspension Test

Following standard pre‐test conditions (including regular circadian rhythm, lighting, and habituation procedures), mice were suspended by their tails in an inverted position using adhesive tape, ensuring minimal discomfort. The test lasted 6 min, during which the duration of immobility—defined as passive hanging without active escape movements—was recorded during the last 5 min.

### 2.22 Forced Swimming Test

Mice were individually placed in a transparent cylindrical container (20 cm diameter, 40 cm height) filled with water at 25°C to a depth that prevented bottom contact or escape. The total test duration was 6 min, and the immobility time—defined as floating with only minimal movements to keep the head above water—was measured during the final 5 min as an indicator of behavioral despair.

### 2.23 Hydrodynamic Tail Vein Injection (HTVI)‐Induced HCC Model

6‐week‐old wild‐type (Cbx5+/+) and knockout (Cbx5–/–) mice were obtained from Schulerbauer Biotechnology Co. Ltd (Wuhan, China). After 1 week of acclimatization, the mice were randomly assigned to two housing conditions: social isolation (SI) or group housing (GH), and maintained under these conditions for 4 weeks. After this period, the mice underwent hydrodynamic tail vein injection (HTVI) to establish a spontaneous HCC model. The injected plasmid mixture included pCAG‐Nras^G12V, pT3‐AKT, and pCMV‐SB100. Following the injection, the mice remained under their respective housing conditions (SI or GH) for an additional 3 weeks until the end of the experiment.

HTVI is a widely used and efficient method for delivering plasmid DNA into hepatocytes in mice. This technique involves the rapid injection of a large volume of DNA solution—approximately 10% of the mouse's body weight—into the tail vein within 5–8 s. The resulting hydrodynamic pressure transiently permeabilizes the membranes of liver cells through a process known as “hydroporation,” thereby facilitating DNA uptake. The high‐pressure infusion causes venous congestion and directs the plasmid solution preferentially to hepatic sinusoids, bypassing systemic circulation and reducing nuclease degradation and immune clearance, thus greatly enhancing transfection efficiency.

To ensure efficient and stable plasmid delivery, several technical parameters were carefully controlled: (1) the injection was completed within 5–8 s to minimize plasmid exposure time in the bloodstream; (2) all plasmids were purified using endotoxin‐free kits to prevent activation of Toll‐like receptors (TLRs) and the associated inflammatory response and DNA degradation; (3) the injection volume was precisely calculated at 10% of the mouse's body weight—for example, a 20 g mouse received 2 mL of plasmid solution—to generate sufficient hydrodynamic pressure to disrupt the endothelial barrier and enhance hepatocyte permeability.

Throughout the experiment, all mice had free access to food and water and were monitored daily for body weight, general condition, and tumor‐related distress. At the end of the study, the mice were euthanized, and liver tissues were collected, weighed, and photographed for further analysis.

### 2.24 Subcutaneous Tumor Xenograft Model

Hep3B cells were resuspended in PBS and mixed 1:1 (v/v) with Matrigel (Corning). A total volume of 100 µL containing 5 × 10^6^ viable cells was injected subcutaneously into the right flank of BALB/c nude mice. Tumor growth was monitored every 2–3 days using calipers. Tumor volume was calculated using the following formula:

$$Volume=1/2\times Length\times Width^{2}$$

Mice were euthanized when the tumor volume reached the predefined endpoint (1500 mm^3^) or at the end of the experimental period. Mice were also euthanized earlier if ulceration or distress occurred. Tumor tissues were excised, weighed, photographed, and processed for subsequent protein extraction, histological analysis, or other experiments.

### 2.25 Taurocholate Treatment

6‐week‐old Cbx5‐WT and Cbx5‐KO mice were randomly assigned into two groups each. After a 1‐week acclimatization period, the Cbx5‐WT + Vehicle and Cbx5‐KO + Vehicle groups were provided with normal drinking water, while the Cbx5‐WT + Taurocholate and Cbx5‐KO + Taurocholate groups received drinking water supplemented with taurocholate (200 mg/kg/day). The taurocholate concentration was adjusted according to average body weight and water consumption to maintain the intended daily dose, and drinking water was refreshed regularly. 6 weeks later, the mice were humanely euthanized, and their livers were collected, measured, weighed, photographed, and fixed in 4% paraformaldehyde or snap‐frozen for downstream assays as appropriate.

### 2.26 Quantification of Taurocholate (TCA) in Plasma and Tumor Tissues

At the experimental endpoint, mice were anesthetized and subjected to cardiac puncture for blood collection. Whole blood was centrifuged at 3000 rpm for 15 min at 4°C to isolate plasma, which was subsequently stored at −80°C for further analysis. Orthotopic liver tumors and subcutaneous tumor tissues were harvested immediately after euthanasia, snap‐frozen in liquid nitrogen, and stored at −80°C until analysis.

The levels of taurocholic acid (TCA) in plasma and tumor tissues were quantified using liquid chromatography–tandem mass spectrometry (LC–MS/MS). Calibration standards and quality‐control samples were included in the same analytical run. Tissue samples were homogenized in pre‐chilled methanol, and processed according to a standardized protocol. TCA levels were normalized to plasma volume or tissue protein concentration. All experiments were performed with at least three biologically independent replicates. Statistical significance was determined using a two‐tailed unpaired t‐test.

### 2.27 Protein‐Protein and Protein‐Ligand Docking Analysis

We obtained the structures of CBX5, c‐Myc, and the small molecule taurocholate using AlphaFold3 and PubChem, respectively. Protein‐protein complex models were generated using AlphaFold3. For CBX5‐taurocholate protein‐ligand modeling, receptor and ligand structures were prepared before docking, and plausible binding poses were selected based on docking scores and visual inspection of interaction geometry. Protein‐protein interfaces were evaluated using PDBePISA, and PyMOL was used to generate structural visualization figures. Docking results were interpreted qualitatively and validated experimentally by pull‐down, co‐immunoprecipitation, and related assays.

### 2.28 Immunohistochemistry (IHC)

Tissue microarray slides (catalog number D097LV01) were purchased from Zhongke Guanghua (Xi'an) Intelligent Biological Technology. The slides were stained using CBX5, PHGDH, Ki67, and AFP antibodies after deparaffinization, rehydration, antigen retrieval, and blocking with an immunohistochemistry (IHC) staining kit (Bios Biological Technology, Wuhan, China). The IHC scores were determined based on the percentage of positively stained cells (0–100%) and the intensity of immunostaining (0 = negative; 1 = weak; 2 = moderate; 3 = strong).

### 2.29 Proximity Ligation Assay (PLA)

Interacting proteins were detected using the Duolink In Situ Red Starter Kit Mouse/Rabbit (DUO92101, Sigma‐Aldrich, Darmstadt, Germany). Hep3B cells were seeded onto coverslips placed in 6‐well plates. After incubation, the cells were gently washed twice with phosphate‐buffered saline (PBS) and fixed with 4% paraformaldehyde for 30 min at room temperature. To quench residual aldehydes, the cells were rinsed with PBS containing glycine and then permeabilized with 0.1% Triton X‐100 in PBS for 20 min. Blocking was performed using a blocking solution composed of PBS, 10% fetal bovine serum (FBS), and 0.1% Triton X‐100. The cells were then incubated overnight at 4°C with anti‐CBX5, and anti‐c‐Myc antibodies diluted in the blocking solution. Following incubation, the slides were washed with 1× Wash Buffer A and incubated with two proximity ligation assay (PLA) probes (diluted 1:5 in Antibody Diluent) for 1 h. This was followed by incubation with ligation solution for 30 min and amplification solution for 100 min at 37°C in a preheated humidified chamber. Prior to imaging, the slides were washed with 1× Wash Buffer B and mounted with coverslips using Duolink In Situ Mounting Medium containing DAPI. Fluorescent images were acquired using a Zeiss LSM 510 Meta confocal microscope. Negative controls omitting one primary antibody were included, and PLA puncta were quantified per cell when required.

### 2.30 Measurement of ALT, AST, CRE, BUN in Mice

Blood was collected from the mouse orbital sinus and stored in heparinized 2 mL centrifuge tubes. The blood and heparin were mixed thoroughly, kept on ice, and centrifuged within 30 min. Subsequently, the samples were centrifuged at 3000 rpm for 15 min at 4°C, and the supernatant was collected as the plasma sample for the assays. Following the kit instructions and sampling procedures, plasma samples and standards from each group of mice were sequentially added to determine the levels of alanine aminotransferase (ALT; Nanjing JianCheng Bioengineering Institute, #C009‐2‐1), aspartate aminotransferase (AST; #C010‐2‐1), creatinine (Cre; #C011‐2‐1), and blood urea nitrogen (BUN; #C013‐2‐1).

### 2.31 PROTAC

Based on established research demonstrating the specific interaction between CBX5 and histone H3, we selected the trimethylated QTARK(Me3)T peptide motif derived from the H3 structure as the protein‐of‐interest (POI) ligand, guided by PDB structural data and our preliminary studies. Given the predominant nuclear localization of CBX5, the E3 ubiquitin ligases von Hippel‐Lindau (VHL) and cereblon (CRBN), both constitutively expressed within the nucleus, were chosen for targeted protein degradation. Consequently, we utilized established ligand binding moieties commonly employed in PROTAC designs to recruit these specific E3 ligases: the VHL ligand VH032 (CAS No.: 1448188‐62‐2) and the Cereblon ligand Pomalidomide (CAS No.: 19171‐19‐8). To ensure adequate cell permeability, linker length was constrained to avoid excessive elongation. Balancing synthetic tractability and minimization of steric hindrance, we designed two distinct linker systems. Integrating these components (POI ligand, E3 ligand, linker), we generated multiple PROTAC candidates encompassing four distinct molecular designs (Figure S2). Synthetic attempts revealed that design (c) afforded the highest synthetic yield and exhibited superior stability among the series. Therefore, based on these findings and consultation with our synthetic chemistry collaborator, design (c) was selected as the optimal construct for further development.

### 2.32 CUT&Tag

The CUT&Tag assay was performed at Jiayin Biotechnology Ltd (Shanghai, China) following standard protocols. Briefly, 500 000 cells were washed twice with a buffer containing 20 mM HEPES (pH 7.5), 150 mM NaCl, 0.5 mM spermidine, and protease inhibitors. Each sample was incubated with 10 µL of ConA magnetic beads at room temperature for 10 min to facilitate cell adherence. Primary antibody (1:50) or an appropriate control antibody was then added, and samples were incubated overnight at 4°C. Unbound primary antibody was removed using a magnetic stand, followed by incubation with a 1:100 dilution of the secondary antibody at room temperature for 1 h, including 2–3 washes with the magnetic stand. A 1:100 dilution of the pA‐Tn5 adapter complex was prepared in buffer, added to the cells, and incubated at room temperature for 1 h. After washing, cells were resuspended in tagmentation buffer containing 10 mM MgCl_2_ and incubated at 37°C for 1 h to facilitate tagmentation. DNA was purified using phenol‐chloroform extraction and ethanol precipitation, followed by PCR amplification using a thermocycler. Data analysis began with quality control using FastQC to assess the quality of the sequencing data. High‐quality reads were then aligned to a reference genome using Bowtie2 to ensure accurate mapping. Peak calling was performed using MACS3 to identify enriched DNA–protein interaction regions and pinpoint binding sites. Motif analysis was conducted using HOMER to reveal sequence motifs within the identified peaks, providing insights into potential transcription factor binding sites and regulatory elements. The raw data have been deposited in GEO under accession number GSE282171.

### 2.33 RNA‐seq

Total RNA was extracted from tissue samples and cells using TRIzol reagent. Libraries were constructed with the Illumina TruSeq RNA kit, during which ribosomal RNA (rRNA) was removed before cDNA synthesis and amplification. Subsequently, paired‐end 150 bp sequencing was performed on the Illumina NovaSeq 6000 platform. Data analysis included quality assessment using FastQC, alignment to the reference genome with HISAT2, quantification of gene expression using featureCounts, and differential expression analysis with DESeq2 using Benjamini‐Hochberg adjusted *p* values; adjusted *p* < 0.05 was considered significant unless otherwise specified. The raw data have been deposited in the Gene Expression Omnibus under accession number GSE282171.

## 3 4‐Dimensional Label‐Free Quantitative Proteomics

The experiments were conducted at Shanghai Applied Protein Technology Co., Ltd. Protein extraction was performed using SDT buffer (4% SDS, 100 mM Tris‐HCl, pH 7.6), and protein concentrations were determined using a BCA assay kit. Each sample (20 µg of protein) was mixed with loading buffer, boiled, separated on a 4–20% SDS‐PAGE gel, and stained with Coomassie Blue. The proteins were digested using the FASP method, desalted on C18 cartridges, dried under vacuum, and reconstituted in 40 µL of 0.1% formic acid. LC‐MS/MS analysis was carried out on a timsTOF Pro mass spectrometer (Bruker) coupled to an Evosep One system. Peptides were separated on a C18 reversed‐phase column using a linear acetonitrile gradient at a flow rate of 220 nL/min. The mass spectrometer operated in positive ion mode over an m/z range of 100–1700, utilizing PASEF mode with one MS scan and eight MS/MS scans per cycle, and an exclusion time of 24 s. Raw MS data for each sample were processed using MaxQuant version 1.6.14 for protein identification and quantification. The data have been deposited under accession PRJCA043173.

### 3.1 Untargeted Metabolomics

The experiment was conducted at Shanghai Applied Protein Technology Co., Ltd. Approximately 80 mg of animal tissue (e.g., mouse liver) was rapidly collected, immediately frozen in liquid nitrogen, and stored. Upon thawing, the tissue was placed in a 2 mL centrifuge tube and homogenized with 200 µL of pure water and ceramic beads. Metabolite extraction was performed by adding 800 µL of methanol/acetonitrile (1:1, v/v) to the homogenate. The mixture was centrifuged at 14 000 × g for 20 min at 4°C, and the supernatant was collected and dried in a vacuum centrifuge. For analysis, the dried sample was re‐dissolved in 100 µL of acetonitrile/water (1:1, v/v), centrifuged again, and the resulting supernatant was used for liquid chromatography‐mass spectrometry (LC‐MS) analysis. Quality‐control samples were prepared by pooling aliquots from individual samples and analyzed throughout the run to monitor instrument stability. The LC‐MS analysis was performed using a UHPLC system (e.g., Agilent 1290 Infinity) coupled with a quadrupole time‐of‐flight mass spectrometer (e.g., AB Sciex TripleTOF 6600) and equipped with a 2.1 mm × 100 mm ACQUITY UPLC BEH Amide column. The scan range was set from 60 to 1000 Da. Raw mass spectrometry data were converted to MzXML format using ProteoWizard and imported into XCMS software for peak picking and alignment. Isotope and adduct annotation was carried out with CAMERA, and metabolite identification was conducted through database comparison. After sum normalization, the data were analyzed using the R package ropls for principal component analysis (PCA) and orthogonal partial least squares discriminant analysis (OPLS‐DA), with sevenfold cross‐validation and permutation testing to assess model robustness. Significant differential metabolites were identified based on variable importance in projection (VIP) scores greater than 1 and *p* < 0.05. Pearson correlation analysis was performed to examine relationships between variables. The data have been deposited in the BioG sub (PRJCA043173).

### 3.2 ATAC‐seq

The ATAC‐seq experiments were conducted by Shanghai Jiayin Biotechnology Ltd. After cell collection and lysis, 50 000 nuclei were isolated and subjected to transposition using the Nextera DNA Library Preparation Kit (Illumina) at 37°C for 30 min. The transposed DNA was then purified with the MinElute PCR Purification Kit (Qiagen) and PCR‐amplified using the NEBNext High‐Fidelity PCR Mix. Sequencing was performed on an Illumina NovaSeq 6000 platform in PE150 mode. For quality control, raw reads were processed to remove adapters and filter out low‐quality sequences; Q20, Q30, and GC content were calculated to ensure data integrity. The cleaned reads were aligned to the reference genome using BWA; duplicate reads and low‐quality alignments were removed before peaks were identified with MACS2 using a q‐value threshold of <0.05. The resulting peak data were visualized with IGV to provide clear representations of open chromatin regions across the genome. The raw data have been deposited in the Gene Expression Omnibus under accession number GSE282171.

### 3.3 Single Cell RNA‐seq

Fresh tissue samples were collected, washed with phosphate‐buffered saline (PBS), and cut into small pieces. The samples were digested with a solution containing collagenase IV, hyaluronidase, and DNase I for 30 min at 37°C, followed by filtration through a 70 µm filter to obtain single‐cell suspensions. Cell viability was assessed before library construction. Single cells were captured using the 10× Genomics Chromium system, encapsulated in microdroplets via microfluidics, and subjected to reverse transcription and PCR amplification for library construction. The libraries were sequenced on the Illumina NovaSeq 6000 platform with 150 bp paired‐end reads, generating approximately 50 000 reads per cell. Data processing included demultiplexing, alignment, and expression‐matrix generation using Cell Ranger. Downstream quality control, clustering, marker‐gene identification, and UMAP visualization were performed using Seurat. The raw data have been deposited in the Gene Expression Omnibus under accession number GSE282171.

### 3.4 Enrichment Analysis

For all sequencing results, we used the R package clusterProfiler to perform enrichment analyses—including KEGG, GO, and GSEA—and for data visualization. Utilizing TCGA‐LIHC data, we analyzed differences in the expression of target genes by dividing samples into high and low expression groups. The association of these genes with various biological pathways was examined through GSEA enrichment analysis of the differentially expressed genes.

### 3.5 Statistical Analyses

For cohort analyses, missing values of covariates were handled using multiple imputation [35]. To assess the association between depression and risk of liver cancer, we used Cox proportional hazard models in the CHARLS and UKB studies, and Logistic regression models in the NHANES. In these analyses, we adjusted for age, sex, race and ethnicity, socioeconomic factors (e.g., education, marital status, living standard, or family income), lifestyle factors (e.g., physical activity, and diet quality assessed by HEI), and health conditions (e.g., BMI, hypertension, hyperlipidemia, and HBV/HCV infection). Further, we stratified the analyses by sex, and likelihood ratio test was used to assess the statistical significance of the interaction. We also conducted a sensitivity analysis by additionally adjusting for antidepressant therapy.

For experimental data, two‐tailed Student's t tests were used for single comparisons, and one or two‐way ANOVA with post hoc tests for multiple comparisons. Data distribution and variance assumptions were checked before parametric testing; non‐parametric tests were used when assumptions were not met. R software (v4.3.1) and GraphPad Prism (v9.0) were used for statistical analyses. *P* < 0.05 was considered statistically significant. All values are expressed as mean ± standard deviation (SD).

## 4 Result

### 4.1 Characteristics of Study Populations

The mean age of participants was 58.5 (9.9) years in CHARLS (n = 14 770), 49.9 (17.7) years in NHANES (n = 29 983), and 56.5 (8.1) years in the UK Biobank (n = 447 748). Non‐Hispanic White participants accounted for 41.6% in NHANES and 95.4% in the UK Biobank. Across the three cohorts, participants with depression were more likely to be female and to have lower socioeconomic status and education level. Cohort‐specific patterns were also observed. In CHARLS, depressed participants were older and had lower BMI and higher alcohol consumption. In NHANES, they were more likely to be current smokers and to have lower physical activity and diet quality. In the UK Biobank, they were more likely to be younger, current smokers, less physically active, and to have higher BMI (Table 1).

**TABLE 1:** Characteristics According to depression degree.

	Depression			
	Overall	No	Mild	Moderate to severe
**CHARLS**				
**CESD‐10 score**	0‐30	≤4	5‐9	≥10
**Number of participants**	14 770	4854	4398	5518
**Age (years)**	58.5 (9.9)	57.3 (9.5)	58.4 (9.8)	59.6 (10.1)
**Sex, %**				
Female	52.7	44.5	50.8	61.4
Male	47.3	55.5	49.2	38.6
**Education, %**				
Illiteracy	44.2	32.6	42.6	55.6
Primary school	10.1	15.2	10.0	5.7
Middle school	22.5	22.1	23.6	22.1
High school or above	23.2	30.1	23.9	16.6
**Living standard, %**				
Poor	12.4	5.7	9.3	20.7
Low	31.5	25.5	33.1	35.6
Average	53.1	64.3	54.7	42.1
High	2.9	4.5	3.0	1.6
**Physical activity, %**				
No	62.0	61.3	61.6	62.8
Low	10.2	10.1	10.5	10.1
Moderate	12.9	13.8	12.7	12.3
Vigorous	14.9	14.8	15.2	14.7
**Smoking, %**				
No	60.9	56.7	60.5	64.9
Yes	39.1	43.3	39.5	35.1
**Alcohol drinking, %**				
Never	24.7	29.1	25.7	20.0
≤ 1 time/month	7.7	8.8	7.7	6.6
> 1 time/month	67.6	62.1	66.6	73.4
**BMI (kg/m^2^)**	23.5 (3.8)	23.9 (3.7)	23.6 (3.8)	23.1 (3.8)
**NHANES**				
**PHQ‐9 score**	0‐27	≤4	5‐9	≥10
**Number of participants**	29 983	22 411	4794	2778
**Age (years)**	49.9 (17.7)	50.0 (17.9)	49.6 (17.8)	49.6 (16.3)
**Sex, %**				
Female	50.8	47.6	58.3	63.6
Male	49.2	52.4	41.7	36.4
**Race, %**				
Non‐Hispanic White	41.6	41.3	42.3	42.1
Non‐Hispanic Black	21.4	21.4	21.7	21.4
Hispanic	10.4	9.9	11.0	13.9
Mexican American	15.0	15.0	15.2	14.8
Others	11.6	12.5	9.8	7.8
**Education, %**				
Less than high school	10.0	9.2	10.5	14.9
High school	28.7	26.7	33.1	36.4
More than high school	61.4	64.0	56.4	48.7
**Income poverty ratio**	2.5 (1.6)	2.7 (1.6)	2.2 (1.5)	1.7 (1.4)
**Smoking status, %**				
Never	55.3	58.2	50.1	40.5
Former	24.3	24.5	24.2	22.5
Current	20.4	17.3	25.7	36.9
**Alcohol drinking, %**				
Never	14.6	15.1	13.3	13.2
Former	15.5	14.5	16.9	21.0
Mild	34.1	36.1	30.1	25.4
Moderate	15.4	15.3	15.9	15.5
Heavy	20.3	19.0	23.8	24.9
**Physical activity, %**				
No	52.6	48.9	59.5	71.0
Moderate	25.7	27.1	23.0	18.6
Vigorous	21.7	24.1	17.4	10.4
**HEI**	54 (13)	55 (14)	53 (13)	51 (13)
**BMI (kg/m^2^)**	29.3 (7.0)	28.9 (6.6)	30.3 (7.8)	31.1 (8.4)

	Depression			
	Overall	No	Yes	
**UKB**				
**PHQ‐4 score**	0‐12	<6	≥6	
**Number of participants**	447 748	422 150	25 598	
**Age (years)**	56.5 (8.1)	56.7 (8.1)	54.0 (7.8)	
**Sex, %**				
Female	54.0	53.7	59.3	
Male	46.0	46.3	40.7	
**Ethnicity, %**				
White	95.4	95.8	88.6	
Others	4.6	4.2	11.4	
**Education, %**				
University	40.5	40.8	34.5	
Others	59.5	59.2	65.5	
**TDI**	−1.4 (3.0)	−1.47 (3.0)	0.06 (3.6)	
**Smoking status, %**				
Never	54.7	55.1	48.8	
Former	34.9	35.2	30.3	
Current	10.4	9.7	20.9	
**Drinking status, %**				
Never	4.1	3.8	7.7	
Former	3.5	3.3	8.0	
Current	92.4	92.9	84.3	
**Physical activity (MET‐minutes /week)**	2651 (2,703)	2667 (2,695)	2385 (2820)	
**HEI**	49 (9)	49 (9)	49 (9)	
**BMI (kg/m^2^)**	27.4 (4.8)	27.3 (4.7)	28.8 (6.0)	

Data expressed as mean [SD] or percentage. Abbreviations: CHARLS, China Health and Retirement Longitudinal Survey; NHANES, National Health and Nutrition Examination Survey. UKB, UK Biobank. PHQ‐9, 9‐item patient health questionnaire; CESD‐10, 10‐item center for epidemiologic studies depression scale; BMI, body mass index; CVD, cardiovascular disease; HEI, healthy eating index. PHQ‐4, 4‐item patient health questionnaire; TDI, Townsend deprivation index; MET, metabolic equivalent task.

### 4.2 The Association of Depression With Liver Cancer

**FIGURE 1:**
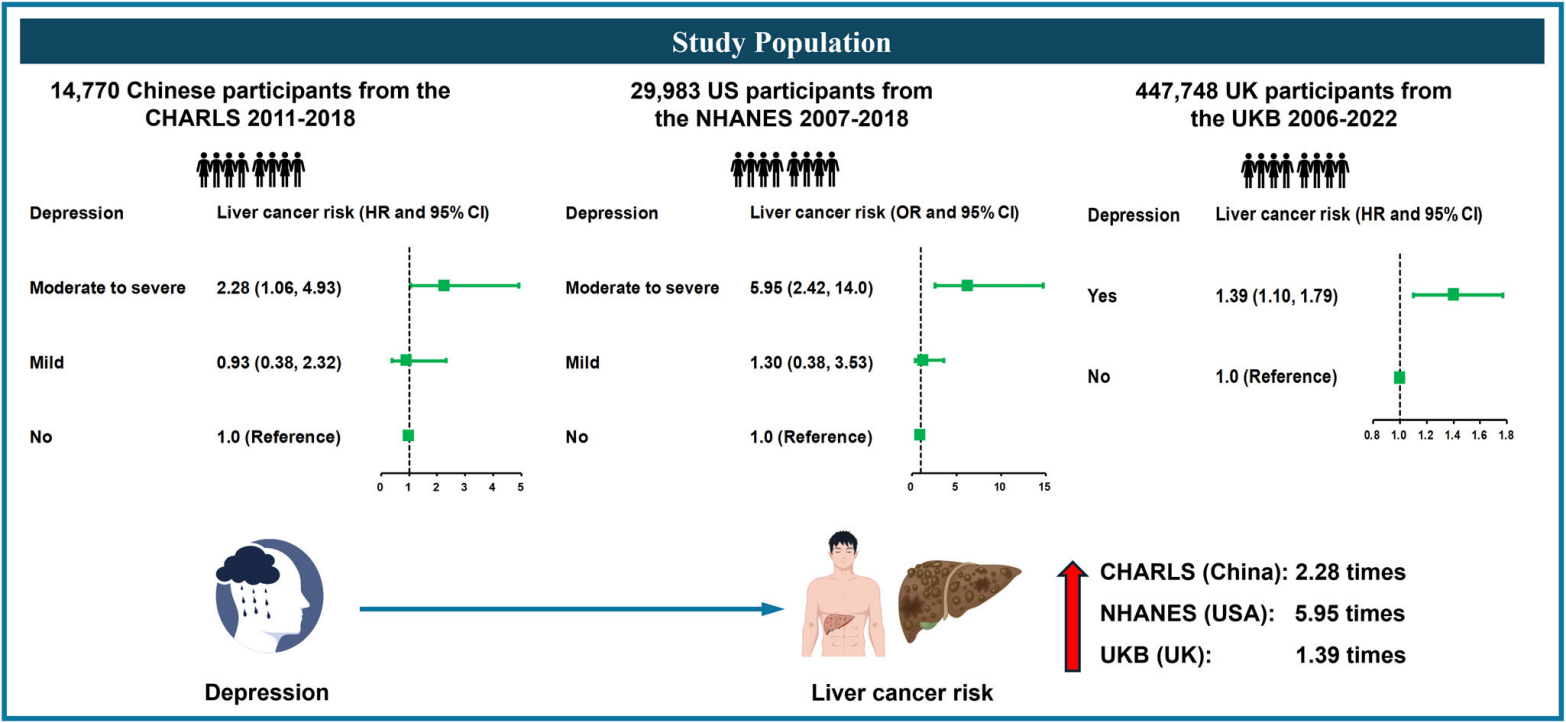
The association between depression and liver cancer. Cox proportional hazard models were used to calculate the HR and 95%CI in CHARLS and UKB, and Logistic regression models were used to calculate the OR and 95%CI in NHANES. Multivariable analyses were adjusted for age, sex, race and ethnicity (NHANES and UKB only), education level, marital status, economic status, physical activity, alcohol drinking status, smoking status, healthy eating index (HEI, NHANES and UKB only), BMI, and history of chronic diseases (e.g., hepatitis B/C virus). CHARLS, China Health and Retirement Longitudinal Study; NHANES, National Health and Nutrition Examination Survey; UKB, UK Biobank. BMI, body mass index; HEI, healthy eating index.

We assessed the association between depression and common cancers and found a consistent positive association between depression and liver cancer risk across three population‐based cohorts (Table S1). In CHARLS (n = 14 770), 46 participants developed liver cancer during a median follow‐up of 7.0 years. In NHANES (n = 29 983), 22 liver cancer cases were recorded. In the multivariable‐adjusted model, compared with the participants without depression, those with moderate to severe depression had a substantially higher risk of liver cancer. The HR and 95% CI comparing moderate to severe depression with no depression were 2.28 (1.06, 4.93) in CHARLS, and the OR and 95% CI were 5.95 (2.42, 14.0) in NHANES (Figure 1) (Table S2). In UKB (n = 447 748), 981 liver cancer cases were recorded during a mean follow‐up of 14.0 years. Compared with participants without depression, those with depression had a 39% (95% CI: 10%–79%) higher risk of liver cancer (Figure 1) (Table S2).

Further, we implemented stratified analyses based on sex subgroups. The association between depression and liver cancer risk did not differ significantly between male and female (*P* interaction > 0.05) (Table S3). Finally, we performed sensitivity analyses with further adjustment for antidepressant therapy. The results remained similar: CHARLS (HR = 2.42, 95% CI: 1.08, 5.39), NHANES (OR = 5.72, 95% CI: 2.43, 13.0), UKB (HR = 1.38, 95% CI: 1.09, 1.78) (Table S4).

### 4.3 Psychological Stress Promotes HCC

**FIGURE 2:**
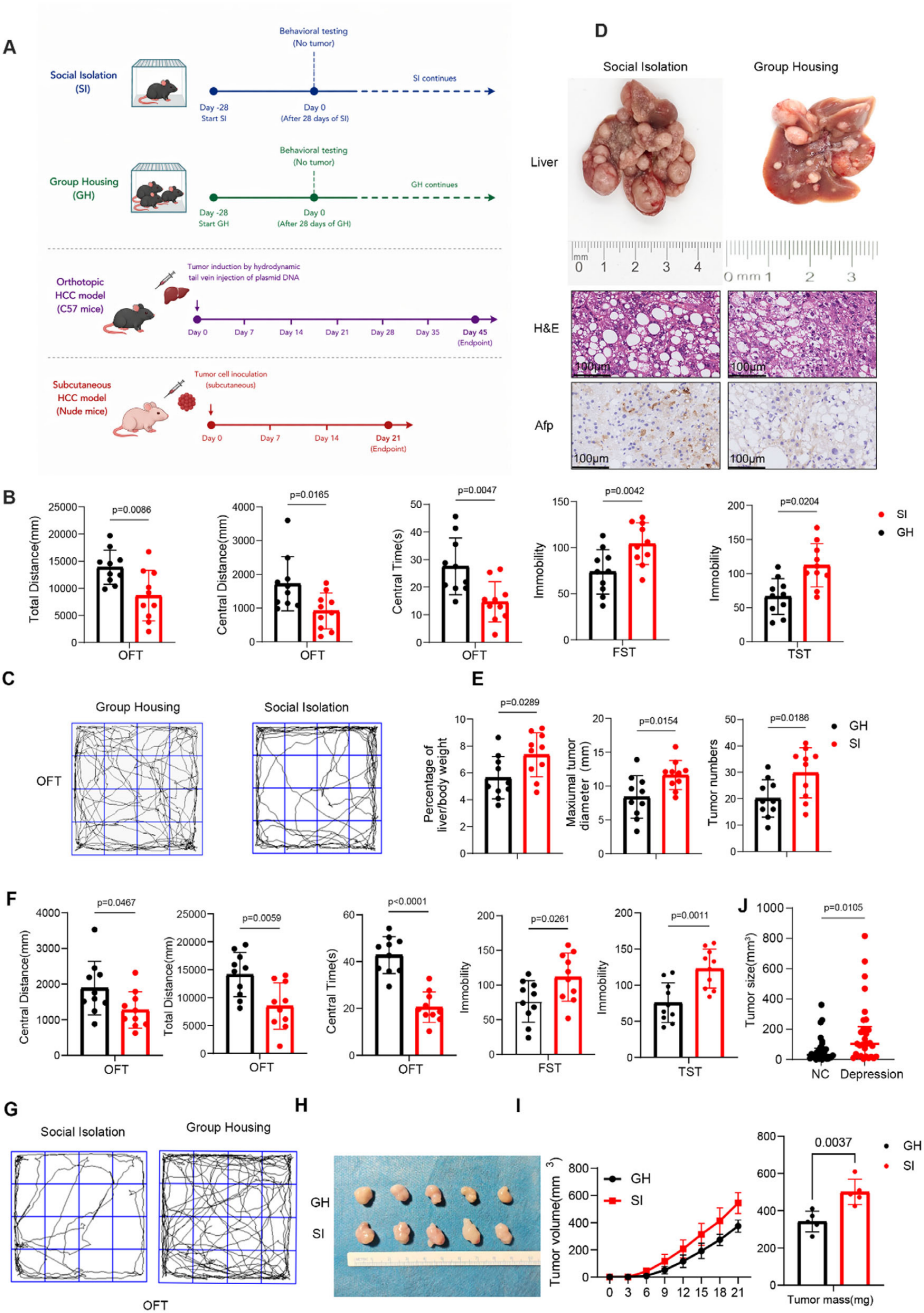
Psychological stress promotes HCC. (A) Schematic diagram of orthotopic and subcutaneous HCC models established in socially isolated (SI) and group‐housed (GH) mice. Experimental timeline is shown below. (B) Behavioral performance in open field test (OFT), forced swim test (FST), and tail suspension test (TST) (n = 10 per group). (C) Representative movement trajectories in the OFT for SI and GH mice. (D) Representative liver images, H&E staining, and AFP immunohistochemistry from orthotopic HCC models in SI and GH mice. (E) Quantification of liver‐to‐body weight ratio, maximum tumor diameter, and tumor number in orthotopic HCC models (n = 10 per group). (F) Behavioral performance in OFT, FST, and TST for subcutaneous HCC models. (G) Representative movement trajectories in the OFT for subcutaneous HCC models. (H) Representative tumor images from subcutaneous Hep3B HCC models. (I) Tumor mass and tumor volume in subcutaneous models (J) Tumor size comparison between HCC patients with depression (n = 30) and without depression (n = 30).

To experimentally evaluate the population‐level association, we established orthotopic and subcutaneous HCC models in mice maintained under GH or SI conditions [17, 36, 37, 38, 39] (Figure 2A). Consistent with previous reports, SI male mice exhibited depression‐like behaviors, including reduced total movement and center‐zone exploration in the open field test, as well as increased immobility in the forced swim and tail suspension tests [39, 40, 41] (Figure 2B,C and F,G). SI also increased HCC burden in mice (Figure 2D,E and H,I). In the human cohort, patients with depression had larger tumors than those without depression (Figure 2J).

### 4.4 Taurocholate Is Elevated in Psychological Stress‐Associated HCC

**FIGURE 3:**
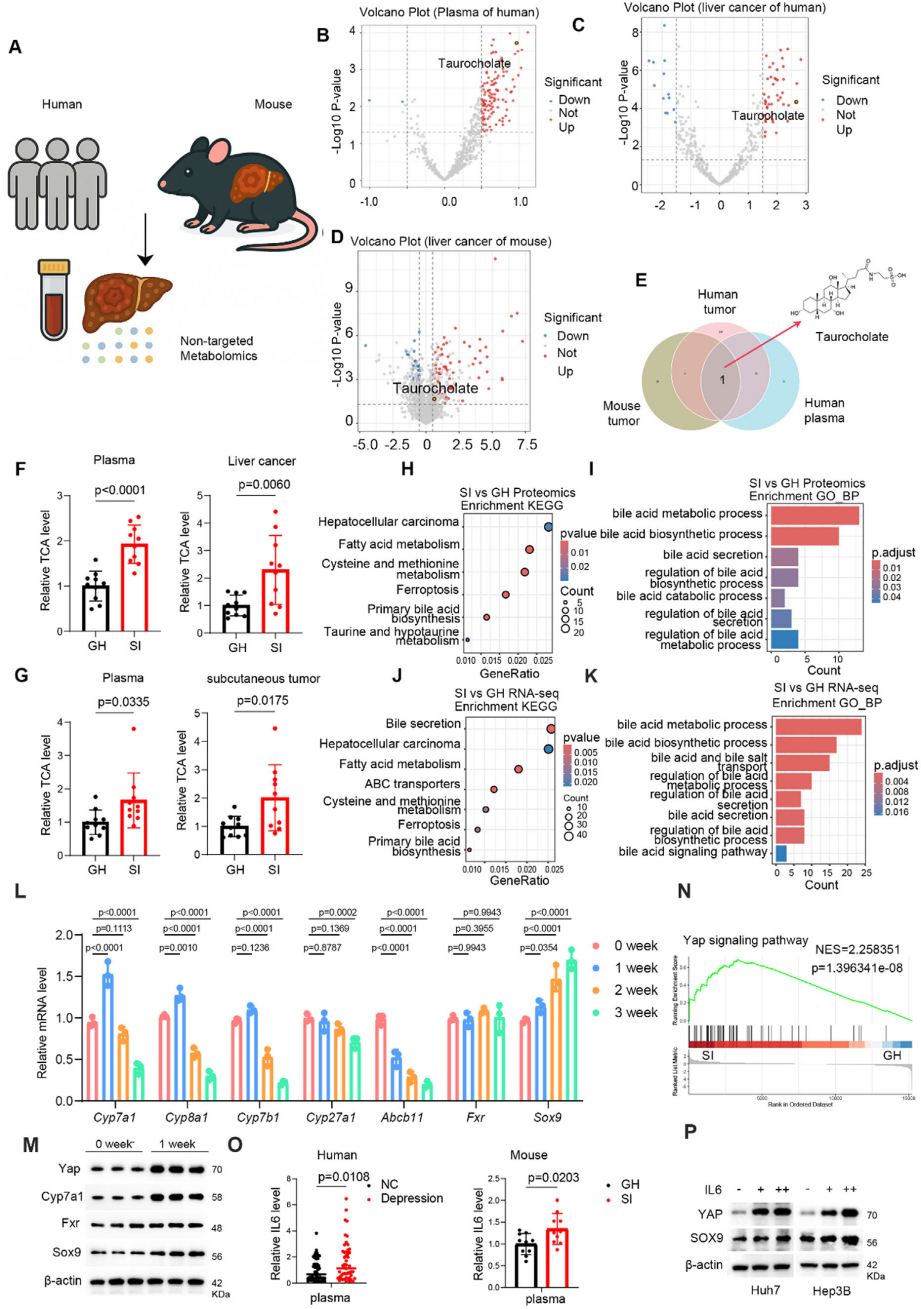
Taurocholate levels were elevated in psychological stress‐associated HCC. (A) Schematic diagram of non‐targeted metabolomics profiling. Plasma and tumor samples were collected from HCC patients with or without depression, and from orthotopic HCC mice under social isolation (SI) or group housing (GH). (B–D) Volcano plots showing differential metabolites between depression and control groups from plasma (B, n = 50/group), tumor tissues (C, n = 15/group), and mouse orthotopic tumors (D, n = 3/group). (E) Venn diagram showing the overlap of upregulated metabolites in human plasma, human tumor, and mouse tumor tissues, highlighting taurocholate. (F,G) Quantification of TCA levels in plasma and tumor tissues from orthotopic (F) and subcutaneous (G) HCC mouse models (n = 10/group). (H,I) KEGG (H) and GO_BP (I) enrichment of differentially expressed proteins from SI and GH HCC mouse tumors based on proteomics (n = 3/group). (J,K) KEGG (J) and GO_BP (K) enrichment of differentially expressed genes from RNA‐seq of SI and GH tumors (n = 3/group; log2(|fold change|) > 1, *p* < 0.05). (L) RT‐qPCR analysis of bile acid metabolism–related genes in orthotopic tumors at different time points (n = 3/group; two‐way ANOVA). (M) Western blot analysis of Cyp7a1 and Fxr in orthotopic tumors at 0 and 1 week. (N) GSEA showing enrichment of Yap signaling in SI versus GH tumors. (O) Plasma IL‐6 levels in HCC patients with or without depression (n = 30/group) and mice in SI versus GH mice (n = 10/group). (P) Western blot analysis of YAP and SOX9 expression in Hep3B and Huh7 cells treated with IL‐6.

Extensive studies have confirmed that circulating metabolites are significantly altered in individuals with depression, thereby influencing tumor initiation and progression [42, 43, 44]. To uncover key metabolic mediators driving psychological stress‐associated HCC progression and to bridge clinical observations with experimental models, we performed untargeted metabolomic profiling on liver tumor and plasma samples from HCC patients with or without depression, as well as on liver tissues from orthotopic HCC mouse models with SI or GH (Figure 3A). Through differential analysis and cross‐species comparison, taurocholate (TCA) emerged as a significantly upregulated metabolite in both humans and mice (Figure 3B–E). Consistently, elevated TCA levels were also observed in the plasma of orthotopic models and in both tumor tissues and plasma from subcutaneous HCC models (Figure 3F,G). These findings align with a recent metabolomic study comparing plasma from patients with major depressive disorder and healthy individuals [45]. As a key component of bile acids, TCA plays a fundamental role in maintaining hepatic metabolic homeostasis [46, 47]. Previous studies have shown that bile acid accumulation can induce depressive symptoms, and recent evidence highlights TCA as one of the most markedly elevated bile acids in cholestatic HCC [48, 49, 50]. Based on these findings, we focused on elucidating the functional role of TCA in psychological stress‐related HCC using mouse models. Transcriptomic and proteomic analyses revealed that differentially expressed genes and proteins were significantly enriched in pathways related to bile acid secretion and regulation (Figure 3H–K). However, key genes involved in bile acid synthesis and export, such as Cyp7a1 and Abcb11, were markedly downregulated at the transcriptional level (Figure S3A). Although paradoxical in the face of an overall rise in bile‐acid levels, our data—consistent with previous studies—demonstrate that depression can induce lineage plasticity in HCC [50, 51, 52]. Mechanistically, after a transient surge, transcripts involved in bile‐acid synthesis (e.g., Cyp7a1) fall sharply, whereas canalicular export genes (e.g., Abcb11) remain persistently repressed. Concurrently, HCC cells activate the YAP‐SOX9 axis and acquire bile‐duct epithelial (BEC)‐like characteristics. This BEC‐like reprogramming reshapes bile‐acid metabolism and drives pathological accumulation of TCA, a change linked to greater tumor heterogeneity, fibrotic stroma, and therapeutic resistance [50, 51, 52]. Our RT‐qPCR and Western blot (WB) analysis at different timepoints further confirmed the dynamic changes in bile acid‐regulatory gene expression (Figure 3L,M). In parallel, transcriptomic analysis revealed activation of YAP signaling in SI‐associated tumors (Figure 3N), consistent with the emergence of a YAP/SOX9‐linked biliary‐like lineage program. We next sought to identify upstream signals that may connect depression‐like stress to activation of this reprogramming axis. Depression has been associated with elevated circulating inflammatory cytokines, particularly interleukin‐6 (IL‐6), a canonical activator of JAK‐STAT signaling [53, 54, 55]. Consistently, IL‐6 levels were significantly increased in both patients with depression and SI mice (Figure 3O), and transcriptomic profiling further showed robust activation of the JAK‐STAT pathway in SI‐associated tumors (Figure S3B). Given previous evidence that IL‐6/STAT3 signaling can induce SOX9 expression and enhance YAP activity [56, 57, 58, 59, 60, 61], we examined this link experimentally and found that IL‐6 treatment upregulated SOX9 and YAP expression in HCC cells (Figure 3P). These results suggest that psychological stress‐associated inflammatory signaling may activate an IL‐6‐JAK/STAT‐SOX9/YAP axis, thereby promoting lineage reprogramming and bile acid dysregulation in HCC.

### 4.5 Taurocholate Inhibits Ferroptosis Through PHGDH in HCC

**FIGURE 4:**
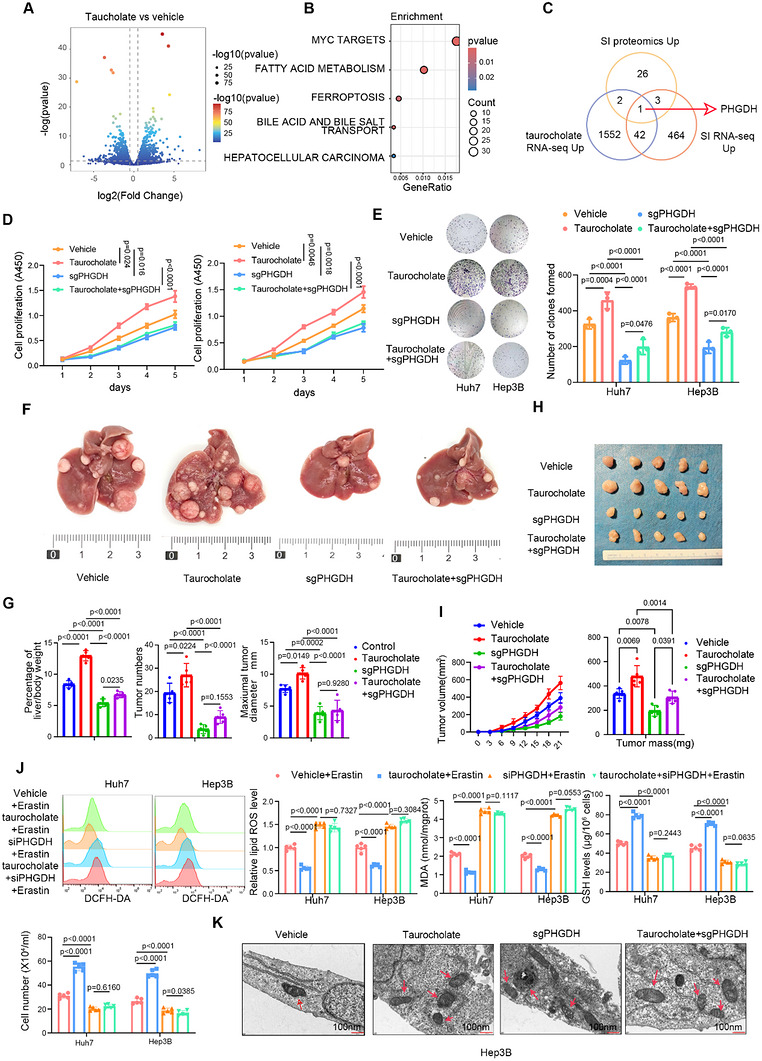
Taurocholate inhibits ferroptosis through PHGDH in HCC (A,B) Transcriptomic analysis of Hep3B cells treated with taurocholate (100 µM). Differentially expressed genes (|log_2_FC| > 1.5, *p* < 0.05) are shown in the volcano plot (A), and enriched pathways of these genes are displayed in (B). (C) Venn diagram showing the overlap between taurocholate‐upregulated genes and the upregulated genes/proteins identified in SI HCC mouse models. (D) CCK‐8 assays of cell proliferation in Hep3B and Huh7 cells transfected with sgPHGDH, with or without taurocholate (100 µM) treatment (n = 3; one‐way ANOVA). (E) Colony formation assays performed in Hep3B and Huh7 cells transfected with sgPHGDH and/or treated with taurocholate (n = 3; two‐way ANOVA). (F) Representative liver images from orthotopic HCC mouse models under the indicated treatments. (G) Quantification of liver‐to‐body weight ratio, tumor number, and maximum tumor diameter in orthotopic HCC mice (n = 5; two‐tailed unpaired *t*‐test). (H) Representative images of subcutaneous Hep3B xenograft tumors under different treatment conditions. (I) Quantification of tumor mass and tumor volume in subcutaneous HCC models (n = 5; two‐tailed unpaired *t*‐test). (J) Ferroptosis‐related assays in cells treated with Erastin (10 µM), siPHGDH, and/or taurocholate, including intracellular ROS detection by DCFH‐DA staining, lipid peroxidation evaluation by MDA and GSH quantification, and cell viability assessment (n = 3; two‐way ANOVA). (K) Transmission electron microscopy showing mitochondrial morphology under different treatments in Hep3B cells.

To define the functional role of taurocholate, we performed transcriptomic sequencing on Hep3B cells treated with taurocholate (Figure 4A). Taurocholate altered ferroptosis‐related pathways (Figure 4B), and similar changes were observed in SI‐associated HCC mouse model (Figure 3H,J). Integration of cell and mouse datasets identified PHGDH as a common differentially expressed gene (Figure 4C), and both SI and taurocholate treatment increased PHGDH expression (Figures S4A,B). Given previous evidence that PHGDH suppresses ferroptosis in prostate and bladder cancers [62, 63], we tested whether PHGDH mediates the effects of taurocholate on HCC cell proliferation. Indeed, TCA significantly promoted HCC cell proliferation, an effect that was partially reversed by PHGDH knockdown (Figure 4D–I). Moreover, taurocholate suppressed ferroptosis, as evidenced by changes in reactive oxygen species (ROS), malondialdehyde (MDA), glutathione (GSH), and mitochondrial morphology (Figure 4J,K).

Together, these results indicate that taurocholate promotes HCC growth, at least in part, by upregulating PHGDH and reducing ferroptotic sensitivity under depressive conditions.

### 4.6 Taurocholate Binds to and Stabilizes CBX5 in HCC

**FIGURE 5:**
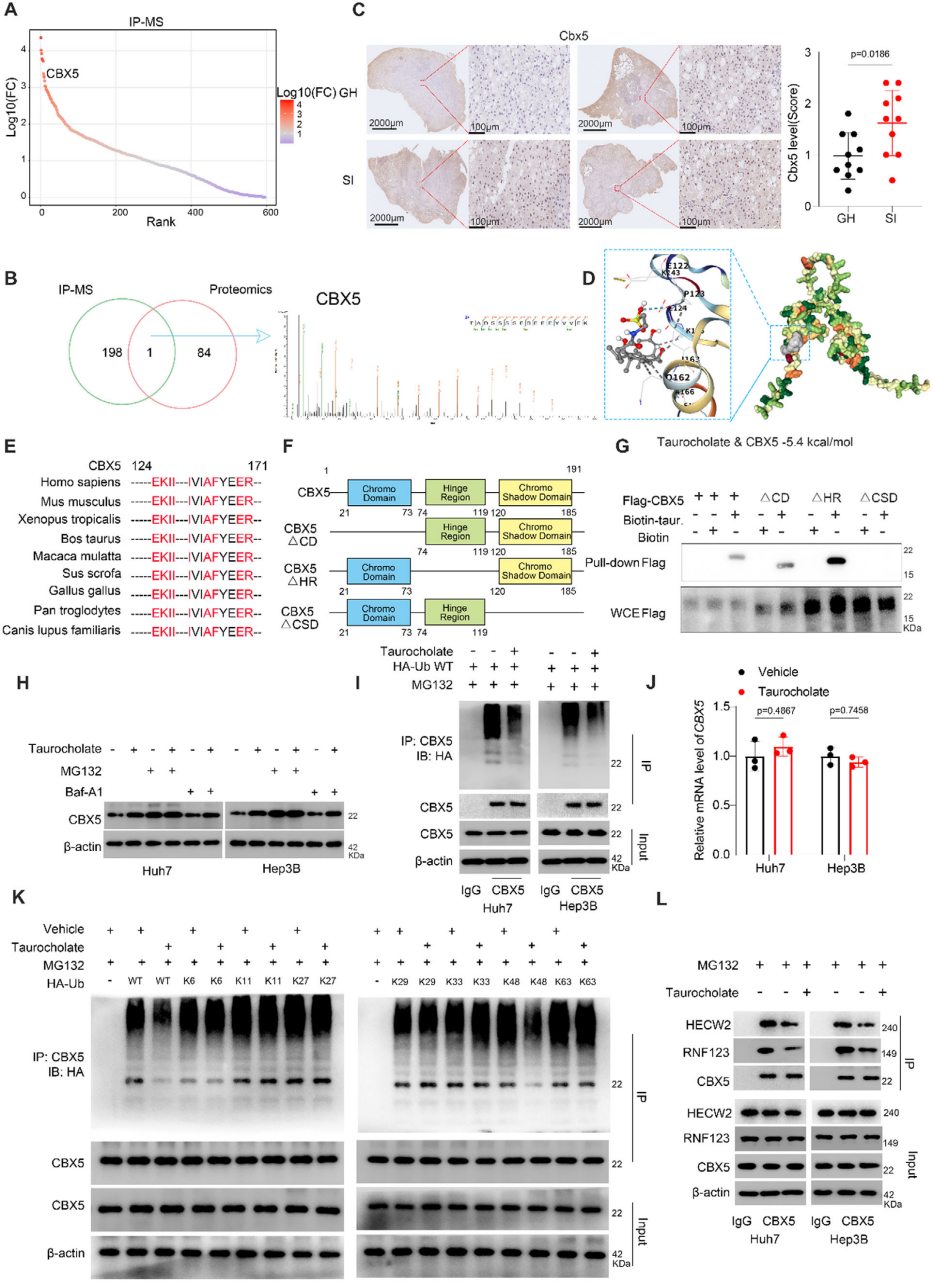
Taurocholate binds to and stabilizes CBX5 in HCC (A) Identification of taurocholate‐binding proteins in Hep3B cells by IP‐MS after treatment with biotin‐taurocholate. (B) Venn diagram showing the overlap between taurocholate‐binding proteins (log_2_FC > 2) and upregulated proteins in orthotopic HCC tumors from SI mice (log_2_FC > 0.6). (C) Immunohistochemical staining of CBX5 in HCC tissues from SI and GH mice (n = 10 per group; two‐tailed unpaired t‐test). (D) Molecular docking model of taurocholate binding to CBX5. (E) Sequence alignment of the taurocholate‐binding region of CBX5 across multiple species showing conserved residues. (F) Schematic diagrams of CBX5 full‐length and deletion mutants: ΔCD (chromodomain deletion), ΔHR (hinge region deletion), and ΔCSD (chromoshadow domain deletion). (G) Flag‐tagged wild‐type CBX5 and its truncation mutants were transfected into Hep3B cells. Cell lysates were incubated with biotin‐labeled taurocholate (biotin‐taur.) or biotin (negative control), followed by pull‐down using streptavidin beads and immunoblotting with anti‐Flag antibody. Upper: Flag‐CBX5 detected in pull‐down samples; Lower: whole cell extract (WCE) input control. (H) Huh7 and Hep3B cells were treated with taurocholate (100 nM), Baf‐A1 (lysosomal inhibitor, 1 µM), or MG132 (proteasome inhibitor, 5 µM), and CBX5 expression was analyzed by western blotting. (I) Cells were co‐transfected with HA‐tagged wild‐type ubiquitin (HA‐Ub WT) and CBX5 plasmids, treated with MG132 and with/without taurocholate, followed by CBX5 immunoprecipitation and detection of ubiquitination using anti‐HA antibody. (J) RT‐qPCR analysis of CBX5 mRNA levels in Huh7 and Hep3B cells treated with or without taurocholate. (K) Huh7 cells were co‐transfected with Flag‐CBX5 and HA‐tagged wild‐type or lysine‐specific ubiquitin mutants (e.g., K6, K11, K27, K29, K33, K48, K63), then treated with MG132 and/or taurocholate. CBX5 was immunoprecipitated and its ubiquitination status was assessed using anti‐HA antibody. (L) Huh7 and Hep3B cells were treated with MG132 and/or taurocholate. Cell lysates were subjected to CBX5 immunoprecipitation and western blotting to examine interactions with different E3 ubiquitin ligases (HECW2, and RNF123).

Although taurocholate is a metabolic molecule, how it upregulates PHGDH expression remains unclear. Given the lack of a direct link between taurocholate accumulation and PHGDH induction, immunoprecipitation‐mass spectrometry using biotin‐labeled taurocholate identified candidate TCA‐interacting proteins (Figure 5A). Intersecting these candidates with proteomic data from SI tumors prioritized CBX5 for further study (Figure 5B).

CBX5, a member of the heterochromatin protein 1 family, has been implicated in several cancers, including lung and breast cancer [64, 65]. Another HP1 family member, CBX3, has been reported to suppress ferroptosis in colorectal cancer via the CUL3/NRF2/GPX2 axis [66]. In our models, immunohistochemistry and western blotting showed increased CBX5 expression in HCC tissues from SI mice compared to GH controls (Figure 5C and Figure S5A), prompting us to examine the taurocholate‐CBX5 interaction.

Molecular docking predicted that taurocholate binds a conserved CBX5 region spanning residues 124–171 (Figure 5D,E). CBX5 truncation experiments further confirmed that this interaction occurs (Figure 5F,G). Functionally, taurocholate stabilized CBX5 protein by suppressing K48‐linked ubiquitination without affecting its mRNA expression (Figure 5H–K and Figure S5B). Taurocholate also weakened the interaction between CBX5 and the E3 ubiquitin ligases RNF123 and HECW2—both previously reported to mediate CBX5 degradation [67, 68], suggesting a mechanism for CBX5 stabilization (Figure 5L).

### 4.7 CBX5 Mediates the Effects of Psychological Stress on HCC

Single‐cell transcriptomic analysis showed that CBX5 is broadly expressed across cell types in murine liver tumors, with the highest expression in HCC cells (Figure S6A,B). Public clinical cohorts further showed that high CBX5 expression was associated with poor prognosis in HCC (Figure S6C,D), and functional assays showed that CBX5 promoted HCC cell proliferation (Figure S6E‐‐H).

Transcriptomic and pathway enrichment analyses linked CBX5 to tumor‐and metabolism‐related pathways, including hepatocellular carcinoma, alcoholic liver disease, fatty acid metabolism, ferroptosis, and peroxisome function (Figure S6I‐‐K), suggesting that CBX5 may connect metabolic reprogramming and cell death processes.

**FIGURE 6:**
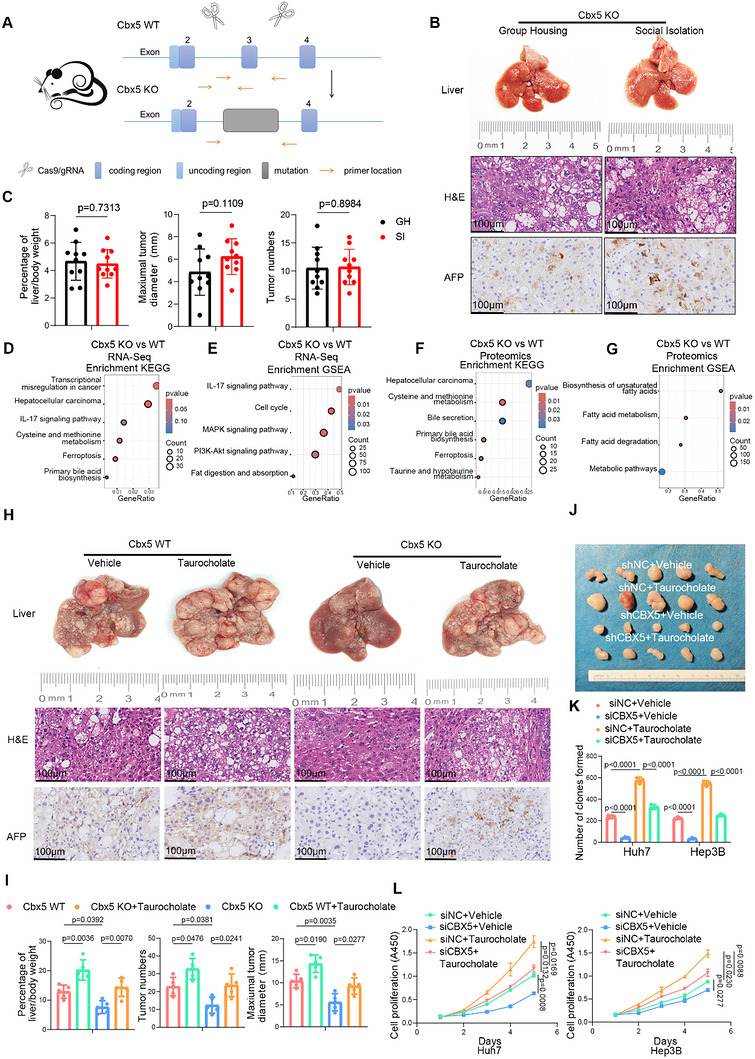
CBX5 Mediates the Effects of psychological stress on HCC (A) Schematic diagram of Cbx5 knockout (KO) mouse construction using CRISPR‐Cas9 targeting exons 2 and 4. (B) Representative liver images, H&E staining, and AFP immunohistochemistry from Cbx5 KO mice housed under group or social isolation conditions. (C) Quantitative analysis of tumor burden in Cbx5 KO mice under SI or GH, including liver‐to‐body weight ratio, maximum tumor diameter, and tumor number (n = 10, two‐tailed unpaired t‐test). (D‐E) RNA‐seq analysis of liver tumors from Cbx5 WT and KO mice (n = 3 per group). KEGG (D) and GSEA (E) enrichment analyses of differentially expressed genes (|FC| > 1, *p* < 0.05). (F,G) Proteomic profiling of liver tumors from Cbx5 WT and KO mice (n = 3 per group). KEGG (F) and GSEA (G) enrichment of differentially expressed proteins (|FC| > 2, *p* < 0.05). (H) Liver gross images, H&E staining, and AFP immunohistochemistry from Cbx5 WT and KO mice treated with vehicle or taurocholate. (I) Quantification of liver‐to‐body weight ratio, tumor number, and maximum tumor diameter in different treatment groups (n = 5 biologically independent mice; one‐way ANOVA). (J) Representative images of subcutaneous tumors formed by Hep3B cells transfected with siNC or siCBX5 and treated with vehicle or taurocholate. (K) Colony formation assays of Hep3B and Huh7 cells transfected with siNC or siCBX5 and treated with or without taurocholate (n = 3, two‐way ANOVA). (L) CCK‐8 assays of Huh7 and Hep3B cells transfected with siRNAs and treated with or without taurocholate (100 µM) for 72 h (n = 3, one‐way ANOVA).

To test the role of CBX5 in HCC progression, we generated a Cbx5 knockout (KO) mouse model (Figure 6A and Figure S7A). Under SI conditions, tumor burden did not increase and remained comparable to that in GH mice (Figure 6B,C and Figure S7B,C), suggesting that CBX5 is required for the full tumor progression.

Integrated multi‐omics analyses on HCC tissues from Cbx5 KO and wild‐type mice showed that Cbx5 deletion affected pathways related to tumor progression, fatty acid metabolism, bile acid metabolism, and ferroptosis (Figure 6D–G), further supporting a role for CBX5 in metabolic and cell fate regulation.

In vitro and in vivo experiments further confirmed the tumor‐promoting effect of taurocholate in a CBX5‐dependent manner (Figure 6H–L and Figure S7D‐‐H). Conversely, implantation of CBX5‐overexpressing tumor cells into GH mice increased tumor growth to levels similar to those observed under SI conditions (Figure S7I‐‐K).

These findings support CBX5 as a key mediator of taurocholate‐driven tumor progression and as a molecular node linking metabolic reprogramming to ferroptosis suppression in psychological stress‐associated HCC.

### 4.8 CBX5 Upregulates PHGDH via MYC in Hcc

**FIGURE 7:**
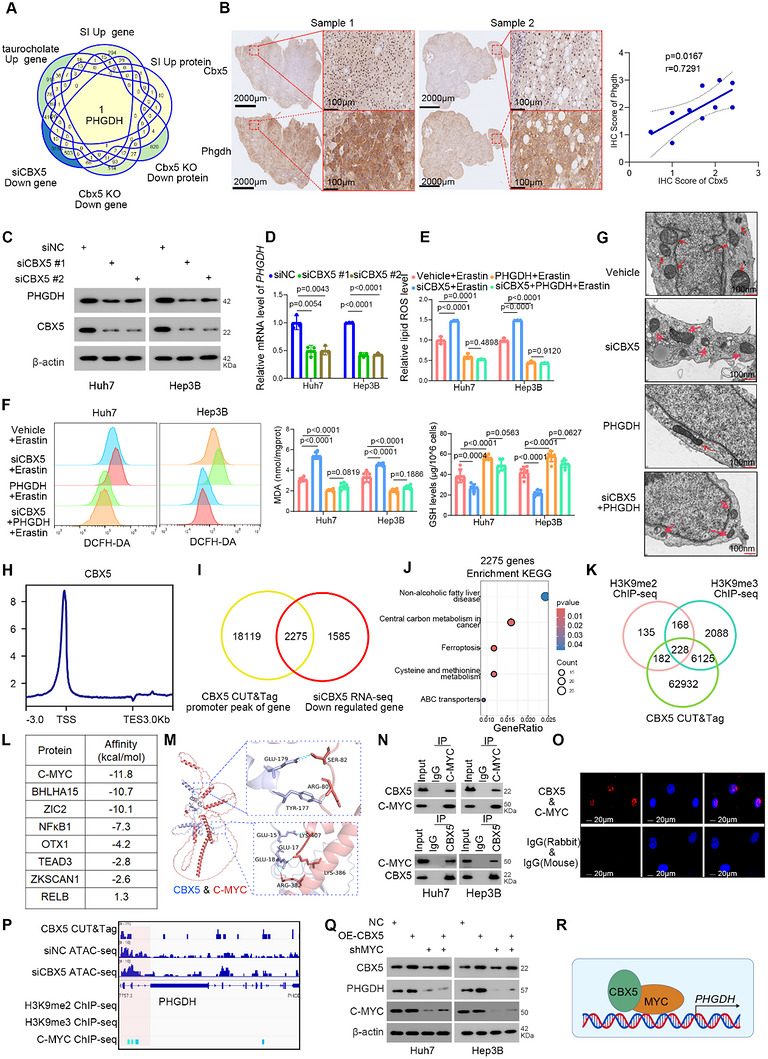
CBX5 upregulates PHGDH via MYC in HCC (A) Venn diagram showing intersecting differentially expressed genes/proteins among multiple datasets, identifying PHGDH as a common downstream target. (B) Immunohistochemical staining of CBX5 and PHGDH in HCC tissues from socially isolated (SI) mice (n = 10). Correlation analysis was performed using Pearson r and unpaired two‐tailed t‐test. (C‐D) Huh7 and Hep3B cells were transfected with siCBX5 for 72 h. PHGDH and CBX5 expression was analyzed by western blotting (C) and RT‐qPCR (D) (n = 3; two‐way ANOVA). (E‐F) Huh7 and Hep3B cells were transfected with siCBX5 or PHGDH plasmids for 72 h and treated with Erastin (10 µM) for 12 h. Intracellular ROS levels were detected by DCFH‐DA staining, and lipid peroxidation levels (MDA, GSH) were quantified (n = 3; two‐way ANOVA). (G) Transmission electron microscopy (TEM) images showing mitochondrial morphology under indicated treatments. (H) CUT&Tag sequencing using CBX5 antibody in Hep3B cells, showing CBX5 enrichment around the transcription start site (TSS). (I) Venn diagram showing overlapping genes between CBX5 CUT&Tag and siCBX5 RNA‐seq downregulated genes. (J) KEGG enrichment of overlapping genes identified in (I). (K) Venn diagram showing overlap among CBX5 CUT&Tag targets, H3K9me2 (from HuH7 ChIP‐seq), and H3K9me3 (from Hep3B ChIP‐seq) genes. (L) Docking affinity scores of CBX5 with transcription factors, showing high affinity with C‐MYC. (M) Structural model of the predicted interaction between CBX5 and C‐MYC proteins. (N) Co‐immunoprecipitation (Co‐IP) confirming the interaction between endogenous CBX5 and C‐MYC in Huh7 and Hep3B cells. (O) Proximity ligation assay (PLA) confirming CBX5 and C‐MYC interaction in Hep3B cells. (P) IGV browser showing PHGDH regulatory region signals from ChIP‐seq, CUT&Tag, and ATAC‐seq data. (Q) Western blot showing the effects of C‐MYC overexpression or knockdown on CBX5‐mediated regulation of PHGDH. (R) Schematic model of CBX5 promoting PHGDH transcription through interaction with C‐MYC.

We next investigated how CBX5 regulates PHGDH. Integrated multi‐omics analysis identified PHGDH as a downstream effector co‐regulated by SI, CBX5, and taurocholate (Figure 7A). PHGDH expression was increased and positively correlated with CBX5 levels (Figure 7B–D and Figure S8A‐‐C). PHGDH overexpression partially rescued the inhibitory effect of CBX5 knockdown on HCC growth (Figure S8D‐‐F).

CBX5 inhibition enhanced ferroptosis, as evidenced by increased ROS and MDA levels, reduced GSH levels, and altered mitochondrial morphology. These effects were partially reversed by PHGDH overexpression (Figure 7E–G and Figure S8G), indicating that CBX5 suppresses ferroptosis partly through PHGDH (Figure S8H).

CBX5 is classically viewed as a transcriptional repressor. It participates in heterochromatin [69]. However, context‐dependent transcriptional activation by CBX5 has also been reported [70]. We therefore examined whether CBX5 activates PHGDH through a non‐canonical mechanism.

CUT&Tag sequencing for CBX5 (Figure 7H and Figure S9A,B). Intersecting promoter‐enriched genes with genes downregulated upon CBX5 knockdown identified 2275 common genes enriched in pathways such as non‐alcoholic fatty liver disease, ferroptosis, and cysteine metabolism (Figure 7I,J). Integration with public H3K9me2/3 ChIP‐seq data from liver cancer showed only partial overlap between CBX5 and H3K9me2/3 binding regions, with non‐overlapping regions mainly located at promoters [71] (Figure 7K and Figure S9C), suggesting non‐canonical mechanisms.

Motif analysis indicated enrichment of transcription factors such as ZKSCAN1 and MYC (Figure 7L and Figure S9D). Protein docking and proximity ligation assays further confirmed an interaction between CBX5 and MYC (Figure 7M‐‐O and Figure S9E,F). ATAC‐seq analysis showed that CBX5 knockdown did not promote chromatin accessibility at the PHGDH promoter (Figure 7P and Figure S10A‐‐C). Because MYC binding sites overlapped with CBX5 binding regions (Figure S10B), we tested MYC dependency and found that CBX5 regulates PHGDH transcription in a MYC‐dependent manner (Figure 7Q and Figure S10D‐‐J). These data suggest that CBX5 can promote PHGDH, by cooperating with MYC (Figure 7R).

In human HCC tissues, CBX5 and PHGDH were higher in patients with depression and were positively correlated (Figure S11A‐‐C). In SI model, time‐course analyses showed sequential increases in TCA, CBX5, and PHGDH (Figure s11D‐‐F). Dose‐response experiments in Huh7 and Hep3B cells further showed that TCA increased CBX5 protein and PHGDH expression, with maximal effects at approximately 100 nM; CBX5 changes occurred earlier than or in parallel with PHGDH induction (Figure S11G‐‐ I).

To further interrogate CBX5 function, we utilized our previously developed H‐PROTAC, which recognizes H3K9me3 and selectively degrades CBX5 or CBX3 [72] (Figure S12A‐‐C). H‐PROTAC did not affect CBX5 transcription but reduced CBX5 and CBX3 protein levels (Figure S12D‐,E). The A‐linker‐PROTAC negative control did not reduce CBX5 protein (Figure S12F,G), and pomalidomide blocked H‐PROTAC–induced CBX5 degradation (Figure S12H).

H‐PROTAC reduced both mRNA and protein levels (Figure S13A), inhibited HCC cell proliferation and promoted ferroptosis in vitro (Figure S13B‐‐D). In vivo, H‐PROTAC suppressed tumor growth in SI mice and enhanced the anti‐tumor efficacy of Erastin (Figure S13E‐‐H). Single‐cell RNA sequencing showed that PROTAC treatment altered pathways related to fatty acid metabolism, oxidative phosphorylation, bile secretion, and MYC targets in HCC cells (Figure S14A‐‐D). Finally, RT‐qPCR confirmed that taurocholate‐induced upregulation was reversed by knockdown of either CBX5 or MYC (Figure S14E).

Together, these findings demonstrate that taurocholate promotes HCC progression by suppressing ferroptosis.

### 4.9 UDCA Attenuates Psychological Stress‐Associated HCC by Reducing Taurocholate

**FIGURE 8:**
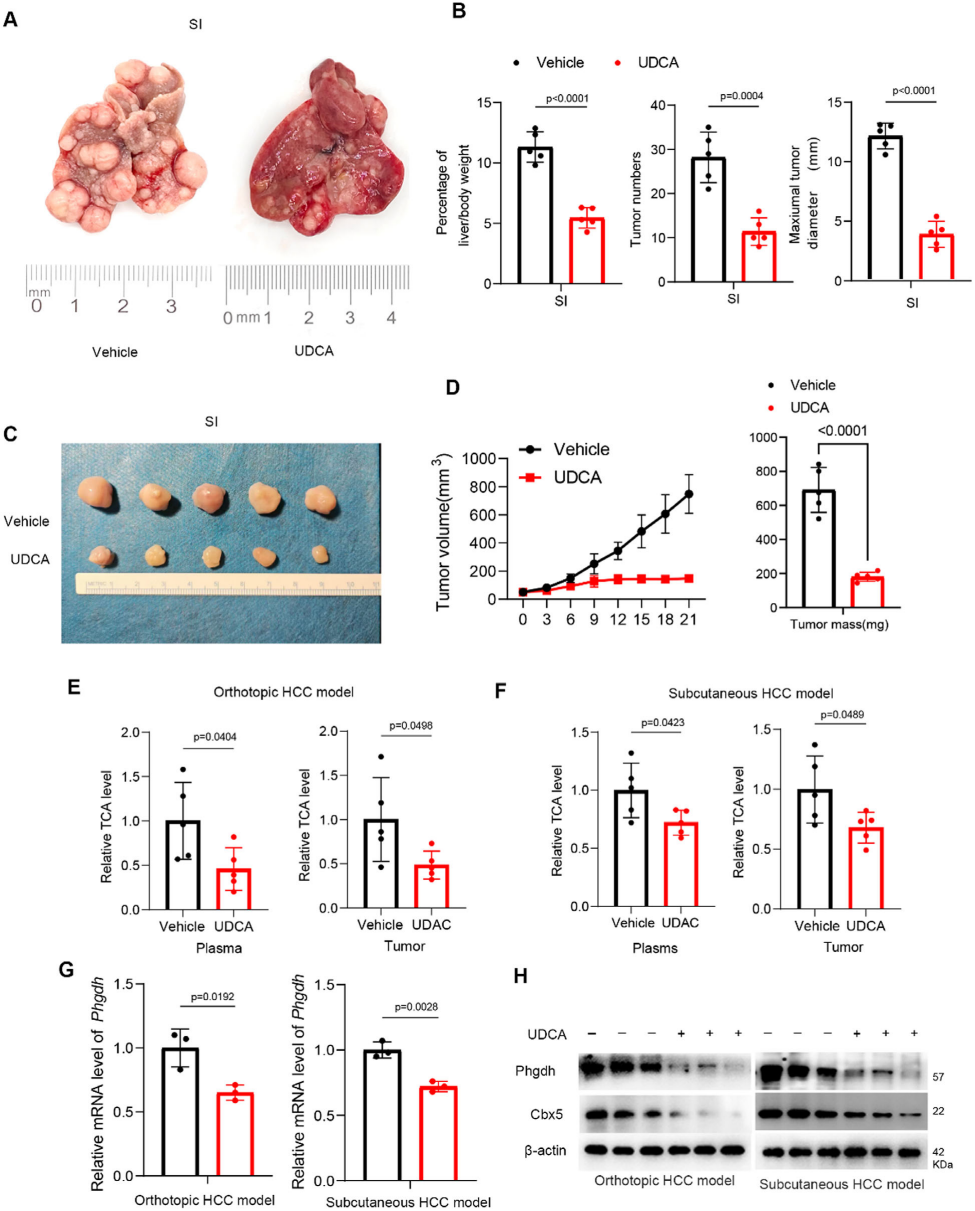
UDCA attenuates psychological stress‐associated HCC by reducing taurocholate (A) Representative liver images from orthotopic HCC mouse models under social isolation (SI) with or without UDCA treatment. (B) Quantification of liver‐to‐body weight ratio, maximum tumor diameter, and tumor number in SI mice treated with or without UDCA (n = 5; two‐tailed unpaired t‐test). (C) Representative tumor images from subcutaneous Hep3B HCC mouse models under SI with or without UDCA treatment. (D) Quantification of tumor mass and tumor volume in subcutaneous HCC models (n = 5; two‐tailed unpaired t‐test). (E) Concentration of TCA in plasma and tumor tissues measured by LC‐MS/MS in orthotopic HCC models (n = 5; mean ± SD; two‐tailed unpaired t‐test). (F) TCA levels in plasma and tumor tissues from subcutaneous HCC models under UDCA treatment (n = 5; mean ± SD; two‐tailed unpaired *t*‐test). (G) Relative mRNA levels of Phgdh in tumor tissues of orthotopic and subcutaneous HCC models treated with or without UDCA, as assessed by RT‐qPCR (n = 3 per group). (H) Western blot analysis of PHGDH and CBX5 protein expression in tumor tissues of orthotopic and subcutaneous HCC models treated with or without UDCA.

Ursodeoxycholic acid (UDCA) is widely used for cholestasis and has been reported to suppress cancer progression [73, 74]. Its role in psychological stress‐associated HCC has not been reported. In orthotopic and subcutaneous HCC mouse models, UDCA reduced tumor progression in depressed mice (Figure 8A–D). UDCA also decreased taurocholate, CBX5 and PHGDH in psychological stress‐related HCC (Figure 8E–H). These findings suggest that UDCA holds promise as a potential therapeutic strategy for psychological stress‐associated HCC.

## 5 Discussion

This study addressed a clinically important but mechanistically unresolved question: whether depression is merely associated with adverse HCC outcomes or whether it may actively contribute to tumor progression through defined biological pathways. By combining three population‐based cohorts with clinical samples, metabolomic profiling, transcriptomic and proteomic analyses, and social isolation models, we found that depression was consistently associated with increased liver cancer risk and that depression‐like stress promoted HCC growth in vivo. Importantly, the experimental data point to bile acid metabolic reprogramming as a mechanistic link between psychological stress and HCC progression.

A major conceptual advance of this work is the identification of taurocholate as a conserved metabolic mediator of psychological stress‐associated HCC. Previous studies have shown that chronic stress or depression can influence tumor biology through neuroendocrine signaling, immune remodeling, gut microbiota changes, and circulating metabolites [75, 76]. Our findings extend this literature by implicating bile acid metabolism, a pathway central to liver physiology, in the tumor‐promoting effects of depressive stress. The consistent elevation of taurocholate in patient samples and mouse models suggests that this metabolite may reflect a disease‐relevant metabolic state rather than a model‐specific observation.

Mechanistically, our data place CBX5 at the center of the taurocholate‐driven response. Taurocholate appears to stabilize CBX5 by weakening its interaction with the E3 ubiquitin ligases RNF123 and HECW2, thereby reducing CBX5 ubiquitination and degradation. This finding is notable because CBX5 is classically viewed as a heterochromatin‐associated transcriptional repressor63. In the present context, however, stabilized CBX5 cooperates with MYC to promote PHGDH transcription. This non‐canonical CBX5‐MYC function provides a plausible route by which a bile acid signal is converted into a transcriptional program that supports HCC cell survival.

The connection between CBX5‐MYC‐PHGDH signaling and ferroptosis further strengthens the biological relevance of this pathway. PHGDH has been reported to suppress ferroptosis in other malignancies [77, 78], and our perturbation experiments show that PHGDH knockdown, CBX5 depletion, CBX5 degradation, or MYC modulation can attenuate taurocholate‐induced tumor‐promoting effects. These results suggest that ferroptosis resistance is an important downstream consequence of psychological stress‐associated bile acid reprogramming. They also provide stronger causal support than association alone, although definitive clinical causality will still require prospective validation.

These findings have several clinical implications. Depression is common among patients with HCC, but it is often treated as a quality‐of‐life issue rather than a potential modifier of tumor biology. Our data suggest that psychological status, bile acid profiles, and ferroptosis‐related markers may deserve closer attention in HCC risk assessment and disease monitoring. In particular, taurocholate, CBX5, and PHGDH may be useful candidates for future biomarker studies designed to identify patients in whom psychological stress‐associated metabolic changes are linked to more aggressive tumor behavior.

The therapeutic implications are also worth considering. UDCA, a clinically used bile acid modulator, reduced taurocholate levels and attenuated tumor progression in social isolation‐associated HCC models, suggesting that bile acid remodeling may be pharmacologically targetable [42]. In parallel, CBX5 degradation and ferroptosis induction suppressed tumor growth in experimental models, raising the possibility that bile acid modulation could be combined with ferroptosis‐based or CBX5‐targeted strategies. However, these results should be interpreted as preclinical evidence. They do not establish UDCA or CBX5 targeting as clinical treatments for psychological stress‐associated HCC, and dedicated translational studies will be required to evaluate efficacy, safety, timing, and patient selection.

Several limitations should be acknowledged. Depression was assessed with different validated instruments across cohorts, which may introduce heterogeneity in exposure definition [43]. Liver cancer ascertainment also differed across datasets, and residual confounding cannot be fully excluded despite adjustment for demographic, socioeconomic, lifestyle, and liver disease‐related factors. In addition, the number of human HCC samples used for mechanistic validation was limited, and social isolation models cannot capture the full complexity of human depression. Future studies should validate this pathway in larger independent clinical cohorts, determine whether taurocholate‐CBX5‐PHGDH markers predict prognosis or treatment response, and test whether psychological intervention or bile acid modulation can alter tumor‐relevant metabolic states. In summary, our study provides an integrated framework in which psychological stress‐associated bile acid reprogramming promotes HCC progression through the taurocholate‐CBX5‐MYC‐PHGDH axis and ferroptosis suppression, highlighting a potentially actionable connection between mental health, metabolism, and liver cancer biology.

## Author Contributions

Methodology: **Ruijiang Zeng**, **Mengmeng Wang**, **Kang Wang**, **Zhuo Xing**, Formal Analysis: **Zhuo Xing**, **Yulong Hong**, **Tongtong Li**, **Geng Zong**, **Chong Yang**, **Kang Wang**, Conceptualization: **Dan Zhang**, **Zhangling Chen**, Investigation: **Dan Zhang**, **Zhangling Chen**, **Xin Jin**, Project Administration: **Xin Jin**.

## Funding

This work was supported by Chinese National Natural Science Foundation (Grant No. 82073321 (X.J.), 82272910 (X.J.), 82473229 (X.J.) 82203814(D.Z)), Excellent Youth Foundation of Hunan Scientific Committee (Grant No. 2022JJ10092, (X. J.)), Hunan Provincial Natural Science Foundation for Young Scholars (Class A) Continuation Project: 2026j20010, Hunan leading program for science and technology innovation of high technology industries (Grant No. 2022GK4020 (X.J.)), Central South University Innovation‐Driven Research Program (Grant No. 2023CXQD058 (X.J.)), Key scientific research project of Hunan Provincial Health Commission (Grant No.W20242004 (X.J.)), and Science and Technology Research Project of Furong Laboratory (Grant No. 2025PT5001 (Z.C.)).

## Conflicts of Interest

Authors declare no competing interests.

## Ethics Statement

This study was conducted in accordance with the Declaration of Helsinki. Ethical approval for patient samples was obtained from the Medical Committee of Sichuan Provincial People's Hospital, University of Electronic Science and Technology of China (Approval No. 2025573‐1). All animal procedures were approved by the Ethics Comechnology Co., Ltd. (Approval No. 2024112601), and all animal experiments were performed in accordance with institutional guidelines for the care and Use of laboratory animals. Patients and Members of the Public Were Not Involved in the Design, Conduct, Reporting, or Dissemination Plans of Our Research

## Clinical Trial Registration

The NHANES study is registered at ClinicalTrials.gov (Identifier: NCT00005154). The UK Biobank and the China Health and Retirement Longitudinal Study (CHARLS) are large‐scale observational cohort studies without clinical trial registration numbers.

## Supporting information




**Supporting File**: advs76416‐sup‐0001‐SuppMat.docx.

## Data Availability

The datasets used and/or analyzed during the current study are available from the corresponding authors on reasonable request.
