# Supplementary material for: Psychological Stress Associated Bile Acid Reprogramming Promotes Hepatocellular Carcinoma Progression
Source: Adv Sci (Weinh). 2026 Jul 11;13(54):e76416. doi: 10.1002/advs.76416 (PMC13355934; doi:10.1002/advs.76416)
Supplement: Supplementary file 1 [file ADVS-13-e76416-s001.docx]

**Psychological Stress Associated Bile Acid Reprogramming Promotes Hepatocellular Carcinoma Progression**

Ruijiang Zeng^1,2,3,4,13^, Mengmeng Wang^5,13^, Kang Wang ^6^^,13^, Zhuo Xing^1,2,3,4,13^, Yulong Hong^1,2,3,4^, Tongtong Li^7^, Geng Zong^7^, Chong Yang^8,9^, Dan Zhang^5*^, Zhangling Chen^10,11,12*^, Xin Jin^1,2,3,4,11,12*^

^1^Department of Urology, The Second Xiangya Hospital, Central South University, Changsha, Hunan 410011, China.

^2^Key Laboratory of Diabetes Immunology (Central South University), Ministry of Education, National Clinical Research Center for Metabolic Disease, Changsha, China.

^3^Uro-Oncology Institute of Central South University, Changsha, Hunan 410011, China.

^4^Hunan Key Laboratory of Tumor Models and Individualized Medicine, The Second Xiangya Hospital, Central South University, Changsha, Hunan 410011, China.

^5^Cancer Center, Union Hospital, Tongji Medical College, Huazhong University of Science and Technology, Wuhan 430022, China

^6^Department of Cardiology, The First Affiliated Hospital of University of South China, Hengyang, PR China.

^7^CAS Key Laboratory of Nutrition, Metabolism and Food Safety, Shanghai Institute of Nutrition and Health, University of Chinese Academy of Sciences, Chinese Academy of Sciences, Shanghai, 200031, China.

^8^Hepatobiliary and Pancreatic Surgery Department, Sichuan Provincial People's Hospital, University of Electronic Science and Technology of China, Chengdu 611731, Sichuan, China;

^9^Chinese Academy of Sciences Sichuan Translational Medicine Research Hospital, Chengdu 610072, Sichuan, China.

^10^Department of Cardiovascular Medicine, The Second Xiangya Hospital of Central South University, Changsha, 410011, PR China

^11^FuRong Laboratory, Changsha, Hunan,410000, China.

^12^Key Laboratory of Cardiometabolic Medicine in Hunan Province.

^13^These authors contributed equally.

***Corresponding authors**: Xin Jin (jinxinxy2@csu.edu.cn), Zhangling Chen (z.chen.1@csu.edu.cn), Dan Zhang (whuh_zd@163.com).

**Figure S1. Participant selection flowchart.**

**Figure S2. PROTAC**

**Figure S3**

(A) Heatmap showing the expression of bile acid metabolism-related genes in liver tissues from social isolation (SI) and group housing (GH) mice.

(B) Gene set enrichment analysis (GSEA) showing enrichment of the JAK-STAT signaling pathway between SI and GH groups.

**Figure S4**

(A) Western blot analysis of PHGDH protein expression in Huh7 and Hep3B cells treated with increasing concentrations of taurocholate.

(B) Western blot analysis of Phgdh protein expression in liver tissues from social isolation (SI) and group housing (GH) mice (n = 3 per group).

**Figure S5**

(A) Western blot analysis of CBX5 protein expression in liver tissues from social isolation (SI) and group housing (GH) mice (n = 3 per group).

(B) Western blot analysis of CBX5 protein stability in Huh7 and Hep3B cells treated with vehicle or taurocholate in the presence of cycloheximide (CHX) for the indicated time points. Relative CBX5 levels normalized to beta-actin are shown in red.

**Figure S6**

(A-B) Single-cell RNA-seq analysis of CBX5 expression in the HCC tumor microenvironment. (A) UMAP plot showing CBX5 expression across cell populations. (B) Violin plots showing CBX5 expression in the indicated cell types.

(C-D) Clinical association of CBX5 expression. (C) Box plots showing CBX5 expression stratified by tumor grade (TCGA-LIHC), BMI (GSE144269), and age (TCGA-LIHC). (D) Kaplan-Meier overall survival analysis according to CBX5 expression in the GSE14520 and GSE144269 cohorts.

(E-F) Huh7 and Hep3B cells were transfected with the indicated siRNAs (E) or plasmids (F) for 72 h, followed by colony formation assays. Representative images and quantification are shown (n = 3 biologically independent experiments; two-tailed unpaired t-test).

(G-H) Cell proliferation was assessed using CCK-8 assays in Huh7 and Hep3B cells transfected with the indicated siRNAs (G) or plasmids (H) for 72 h (n = 3 biologically independent experiments; two-way ANOVA).

(I-K) RNA-seq was performed in Hep3B cells 72 h after siCBX5 transfection. (I) Volcano plot showing differentially expressed genes (|log2FC| > 1, P < 0.05). (J) KEGG enrichment analysis and (K) GSEA of downregulated genes.

**Figure S7**

(A) Western blot validation of CBX5 protein expression in liver, heart, and kidney tissues from Cbx5 wild-type (WT) and knockout (KO) mice (n = 3 per group).

(B) Representative open field test (OFT) movement traces of group-housed (GH) and socially isolated (SI) mice.

(C) Quantification of behavioral parameters in the OFT (total distance, central distance, and central time), forced swim test (FST), and tail suspension test (TST) in GH and SI mice (n = 10 per group; unpaired t-test).

(D) Tumor weight (left) and tumor growth curves (right) in subcutaneous xenograft models treated with vehicle, taurocholate, shCBX5, or the indicated combination (n = 5 per group; one-way or two-way ANOVA).

(E) Representative colony formation assays in Huh7 and Hep3B cells transfected with siNC or siCBX5, with or without taurocholate treatment.

(F) Flow cytometric analysis of intracellular ROS levels using DCFH-DA staining in Huh7 and Hep3B cells treated as indicated in the presence of erastin.

(G-H) Quantification of ferroptosis-associated indicators, including lipid ROS, malondialdehyde (MDA), glutathione (GSH), and viable cell number, in Huh7 and Hep3B cells treated with siCBX5, taurocholate, or their combination in the presence of erastin for 72 h (n = 5; two-way ANOVA).

(I-K) Subcutaneous tumors were established using wild-type or CBX5-overexpressing Hep3B cells in GH or SI mice. Tumor volume and tumor weight were recorded (n = 5 per group).

**Figure S8**

(A) Correlation analysis between CBX5 and PHGDH expression in the TCGA-LIHC dataset.

(B) Western blot analysis of PHGDH and CBX5 expression in liver tissues from Cbx5 wild-type (WT) and knockout (KO) mice (n = 3 per group). Beta-actin was used as a loading control.

(C) Huh7 and Hep3B cells were transfected with Flag-CBX5 or empty vector (EV) for 72 h. Protein and mRNA levels of PHGDH and CBX5 were analyzed by western blotting and RT-qPCR (n = 3; two-tailed unpaired t-test).

(D) Proliferation curves of Huh7 and Hep3B cells after CBX5 knockdown, PHGDH overexpression, or the combined treatment. Proliferation was assessed using CCK-8 assays over 5 days (n = 3; one-way ANOVA).

(E) Representative colony formation assays in Huh7 and Hep3B cells with the indicated CBX5 knockdown and/or PHGDH overexpression treatments.

(F) Quantification of colony numbers in (E) (n = 3; two-way ANOVA).

(G) Cell viability of Huh7 and Hep3B cells treated with erastin in combination with siCBX5 and/or PHGDH overexpression, assessed by cell counting (n = 5; two-way ANOVA).

(H) Schematic model showing that CBX5 promotes ferroptosis resistance through transcriptional upregulation of PHGDH.

**Figure S9**

(A) CUT&Tag profiling of CBX5 in Hep3B cells. The heatmap shows CBX5 signal intensity across ±3 kb regions centered on transcription start sites (TSSs).

(B-C) Pie charts showing the genomic distribution of CBX5 binding peaks annotated to genomic features.

(D) Motif enrichment analysis of CBX5 binding regions using HOMER. Top enriched motifs and associated transcription factors are shown.

(E) Co-immunoprecipitation (Co-IP) showing the interaction between exogenous Flag-CBX5 and HA-MYC in 293T cells.

(F) Quantification of proximity ligation assay (PLA) signals for the CBX5-MYC interaction in Hep3B cells (n = 3 biologically independent experiments; two-tailed unpaired t-test).

**Figure S10**

(A) Line plots showing chromatin accessibility around transcription start sites (TSSs) in siCBX5 and siNC groups based on ATAC-seq clustering.

(B-C) IGV genome browser views showing ChIP-seq, CUT&Tag, and ATAC-seq signals at the PHGDH locus.

(D-F) Huh7 and Hep3B cells were transfected with the indicated constructs for 72 h. Cells were harvested for ChIP-qPCR to assess MYC and CBX5 enrichment at the PHGDH promoter (n = 3 biologically independent experiments; two-tailed unpaired t-test or two-way ANOVA).

(G) Schematic diagram of the PHGDH promoter region and the corresponding wild-type (WT) and mutant (Mut) motif sequences used in luciferase assays.

(H-I) Dual-luciferase reporter assays were performed to assess the transcriptional activity of WT or Mut PHGDH promoters in Huh7 and Hep3B cells transfected with the indicated constructs (n = 3; two-way ANOVA).

(J) Huh7 and Hep3B cells were transfected with the indicated constructs for 72 h. Cells were harvested for RT-qPCR

**Figure S11**

(A) Representative western blot analysis of PHGDH and CBX5 protein levels in HCC tissues from patients with depression (ED, n = 30) and non-depressed controls (NC, n = 30). Tumor size was also assessed. Beta-actin was used as a loading control, and relative protein levels are indicated in red.

(B) Quantification of CBX5 and PHGDH expression levels in ED versus NC HCC tissues based on western blot analysis (n = 30 per group; two-tailed unpaired t-test).

(C) Correlation analysis of CBX5 and PHGDH expression in HCC tissues (n = 30 per group; Pearson correlation and two-tailed unpaired t-test).

(D) Quantification of taurocholate (TCA) levels in liver tumors at the indicated time points (0, 1, 2, and 3 weeks) in the experimental model. Data are presented as mean +/- SEM; statistical significance was determined by one-way ANOVA with Tukey's post hoc test.

(E) Representative western blot analysis of CBX5 protein levels in liver tissues collected at different time points (0-3 weeks). Beta-actin was used as a loading control.

(F) RT-qPCR analysis of Phgdh mRNA expression at different time points (0-3 weeks) in liver tissues. Data are presented as mean +/- SEM; statistical significance was assessed by one-way ANOVA with Tukey's post hoc test.

(G) Representative western blot analysis showing PHGDH and CBX5 protein levels in Huh7 and Hep3B cells treated with increasing concentrations of TCA (0-10^3 nM). Beta-actin was used as a loading control.

(H-I) Quantification of PHGDH and CBX5 protein levels in Huh7 (H) and Hep3B (I) cells following TCA treatment at the indicated concentrations. Data are presented as mean +/- SEM from three independent experiments.

**Figure S12**

(A) Structural model showing the binding of CBX5 to the H3K9(me)3 peptide.

(B) Chemical structure of the designed PROTAC molecule, consisting of an H3K9(me)3 peptide (red), a linker, and a pomalidomide moiety (blue) for E3 ligase recruitment.

(C) Molecular docking model of the H3K9(me)3-based PROTAC bound to CBX5.

(D) Huh7 and Hep3B cells were treated with increasing concentrations of PROTAC for 72 h, followed by RT-qPCR analysis of CBX5 mRNA expression (n = 3).

(E) Western blot analysis of CBX5 and CBX3 protein levels in Huh7 and Hep3B cells treated with increasing concentrations of PROTAC for 72 h.

(F) Chemical structure of a control PROTAC in which the H3K9(me)3 peptide was replaced by a nonspecific AAAAA peptide, with the same linker and pomalidomide components.

(G) Western blot analysis of CBX5 protein levels in Huh7 and Hep3B cells treated with control PROTACs (H-linker or A-linker) at the indicated concentrations.

(H) Western blot analysis of CBX5 expression in Huh7 and Hep3B cells treated with H-linker PROTAC in the presence or absence of excess free pomalidomide (100 uM).

**Figure S13**

(A) Huh7 and Hep3B cells were treated with increasing concentrations of PROTAC (0, 1, and 2 uM) for 72 h. PHGDH and CBX5 protein levels were assessed by western blotting, and PHGDH mRNA levels were measured by RT-qPCR (n = 3).

(B) Cell proliferation was measured using CCK-8 assays in Huh7 and Hep3B cells treated with vehicle, erastin (1 uM), PROTAC (1 uM), or the combination for 5 days (n = 3).

(C) Intracellular ROS levels were analyzed by DCFH-DA staining and flow cytometry. Lipid ROS, malondialdehyde (MDA), and glutathione (GSH) levels were quantified, and cell viability was measured by direct cell counting (n = 3).

(D) Colony formation assays were performed in Huh7 and Hep3B cells treated as indicated for 10 days. Representative images and colony quantification are shown (n = 3).

(E) Subcutaneous tumor models were established using Hep3B cells. Mice were treated with vehicle, erastin, locally injected PROTAC, or the combination for 21 days. Tumor volume and tumor weight were recorded (n = 5 per group).

(F) Orthotopic HCC models were established and treated as indicated for 4 weeks. Representative liver tumor images were collected at the endpoint.

(G) Tumor burden in the orthotopic HCC model was quantified by liver tumor weight-to-body weight ratio, tumor nodule number, and maximum tumor diameter (n = 5).

(H) Serum biochemical markers, including ALT, AST, creatinine (CRE), and blood urea nitrogen (BUN), were measured to evaluate systemic toxicity after local PROTAC administration (n = 5).

**Figure S14**

(A) UMAP plot showing cell clustering of orthotopic HCC tissues treated with vehicle or locally injected CBX5-PROTAC based on single-cell RNA-seq (n = 3 per group).

(B) Pie charts showing the proportions of distinct cell types in HCC tissues from PROTAC and vehicle groups, identified using canonical markers.

(C) Enrichment analysis of HCC cells based on differentially expressed genes between PROTAC and vehicle groups, including hallmark pathway enrichment (left) and KEGG pathway analysis (right).

(D) GO enrichment analysis of differentially expressed genes in HCC cells from the PROTAC-treated group.

(E) RT-qPCR analysis of PHGDH mRNA levels in Hep3B and Huh7 cells transfected with the indicated siRNAs and treated with taurocholate (n = 3 biologically independent experiments; two-tailed unpaired t-test or two-way ANOVA).

**Table S1. The association between depression and common cancers.**

|  | **Lung cancer** | **Stomach cancer** | **Thyroid cancer** | **Colorectal cancer** | **Prostatic cancer** | **Breast cancer** | **Cervical cancer** | **Liver cancer** |
| --- | --- | --- | --- | --- | --- | --- | --- | --- |
| **CHARLS** |  |  |  |  |  |  |  |  |
| No | 1.00 (Ref) | 1.00 (Ref) | 1.00 (Ref) | 1.00 (Ref) | 1.00 (Ref) | 1.00 (Ref) | 1.00 (Ref) | 1.00 (Ref) |
| Mild | 1.18 (0.59, 2.36) | 2.24 (1.01, 4.97) | 0.18 (0.02, 1.53) | 1.05 (0.45, 2.46) | 1.18 (0.16, 8.69) | 0.93 (0.32, 2.66) | 1.38 (0.52, 3.65) | 0.93 (0.38, 2.32) |
| Moderate to severe | 1.12 (0.55, 2.28) | 1.36 (0.58, 3.23) | 0.72 (0.20, 2.54) | 0.64 (0.25, 1.67) | 1.78 (0.26, 12.21) | 1.40 (0.54, 3.59) | 1.84 (0.74, 4.60) | 2.27 (1.05, 4.91) * |
| **NHANES** |  |  |  |  |  |  |  |  |
| No | 1.00 (Ref) | 1.00 (Ref) | 1.00 (Ref) | 1.00 (Ref) | 1.00 (Ref) | 1.00 (Ref) | 1.00 (Ref) | 1.00 (Ref) |
| Mild | 1.11 (0.67, 1.77) | 1.22 (0.29, 3.81) | 1.29 (0.64, 2.44) | 1.35 (0.97, 1.86) | 1.09 (0.87, 1.35) | 1.04 (0.82, 1.29) | 1.23 (0.84, 1.76) | 1.34 (0.39, 3.63) |
| Moderate to severe | 1.05 (0.54, 1.88) | 3.03 (0.90, 8.89) | 1.78 (0.75, 3.79) | 1.83 (1.23, 2.65)** | 1.36 (1.00, 1.82)* | 1.41 (1.07, 1.85)* | 1.34 (0.89, 1.97) | 6.32 (2.59, 14.75) *** |
| **UKB** |  |  |  |  |  |  |  |  |
| No | 1.00 (Ref) | 1.00 (Ref) | 1.00 (Ref) | 1.00 (Ref) | 1.00 (Ref) | 1.00 (Ref) | 1.00 (Ref) | 1.00 (Ref) |
| Yes | 1.20 (1.08, 1.32) *** | 1.28 (1.01, 1.62)* | 1.02 (0.72, 1.46) | 0.95 (0.85, 1.07) | 1.01 (0.91, 1.11) | 1.02 (0.94, 1.11) | 0.93 (0.53, 1.66) | 1.40 (1.10,1.77) * |

Abbreviations: CHARLS, China Health and Retirement Longitudinal Survey; NHANES, National Health and Nutrition Examination Survey; UKB, UK Biobank.

CHARLS: The model was adjusted for sex, age, marital, education, living standard, BMI, smoking, alcohol drinking, physical activity, hypertension, hyperlipidemia, diabetes, CVD and liver disease.

NHANES: The model was adjusted for sex, age, race, marital, education, family income to poverty ratio, BMI, smoking, alcohol drinking, physical activity, and HEI.

UKB: The model was adjusted for age, sex, ethnicity, BMI, education, TDI, smoking, alcohol drinking, physical activity, HEI, hypertension, diabetes, hyperlipidemia and CVD.

^*^*P*<0.05; ^**^*P*<0.01; ^***^*P*<0.001.

**Table S2. The association between depression with liver cancer.**

|  | **Depression** | | |
| --- | --- | --- | --- |
|  | **No** | **Mild** | **Moderate to severe** |
| **CHARLS** |  |  |  |
| Cases/Person-years | 10/31,258 | 9/28,420 | 27/35,333 |
| Model 1, HR (95%CI) | 1.00 (Ref) | 0.99 (0.40, 2.44) | 2.44 (1.17, 5.10) ^*^ |
| Model 2, HR (95%CI) | 1.00 (Ref) | 0.93 (0.38, 2.32) | 2.28 (1.06, 4.93) ^*^ |
| **NHANES** |  |  |  |
| Cases/Participants | 12/22,411 | 3/4,794 | 7/2,778 |
| Model 1, OR (95%CI) | 1.00 (Ref) | 1.25 (0.35, 3.45) | 5.51 (2.32, 12.3) ^***^ |
| Model 2, OR (95%CI) | 1.00 (Ref) | 1.30 (0.38, 3.53) | 5.95 (2.42, 14.0) ^***^ |
|  | **No** | **Yes** |  |
| **UKB** |  |  |  |
| Cases/Person-years | 903/5,893,252 | 78/356,783 | ─ |
| Model 1, HR (95%CI) | 1.00 (Ref) | 1.95(1.54, 2.46) ^***^ | ─ |
| Model 2, HR (95%CI) | 1.00 (Ref) | 1.39 (1.10, 1.79) ^*^ | ─ |

Abbreviations: CHARLS, China Health and Retirement Longitudinal Survey; NHANES, National Health and Nutrition Examination Survey; UKB, UK Biobank.

CHARLS:

Model 1: Age (continuous, years), and sex (female or male).

Model 2: Model 1 + marital status (with and without partner), education level (Illiteracy, primary school, middle school and high school or above), living standard (high, average, low and poor), smoking (no and yes), alcohol drinking (never, ≤1 time/month and >1 times/month), physical activity (no, low, moderate and vigorous), BMI (continuous, kg/m^2^), hypertension (yes, no), hyperlipidemia (yes, no), diabetes (yes, no), CVD (yes, no), and liver diseases including hepatitis B/C infection (yes, no)

NHANES:

Model 1: Age (continuous, years), and sex (female and male).

Model 2 : Model 1 + race and ethnicity (Non-Hispanic White, Non-Hispanic Black, Hispanic, Mexican American and others), marital status (with and without partner), education level (less than high school, high school and more than high school), family income to poverty ratio (continuous), smoking (never, former and current), alcohol drinking (never, former, mild, moderate and heavy), physical activity (no, moderate and vigorous), HEI (continuous), BMI (continuous, kg/m^2^), and liver diseases including hepatitis B/C infection (yes, no)

UKB:

Model 1: Age (continuous, years), and sex (female and male).

Model 2 : Model 1 + race and ethnicity (White and others), marital status (with and without partner), education level (university degree or other), TDI (continuous), smoking (never, former, and current), alcohol drinking (never, former, and current), physical activity (continuous, MET-minutes/week), HEI (continuous), BMI (continuous, kg/m^2^), hypertension (yes, no), hyperlipidemia (yes, no), diabetes (yes, no), CVD (yes, no), and liver diseases including hepatitis B/C infection (yes, no)

^*^P<0.05, **P<0.01, ***P<0.001.

**Table S3. The association between depression and liver cancer in different genders.**

|  | **Depression** | | | ***P*** **_interaction_** |
| --- | --- | --- | --- | --- |
|  | **No** | **Mild** | **Moderate to severe** |  |
| **CHARLS** |  |  |  | 0.12 |
| Female | 1.00 (Ref) | 0.98 (0.14, 6.99) | 5.54 (1.23, 24.9) ^*^ |  |
| Male | 1.00 (Ref) | 0.95 (0.34, 2.69) | 1.44 (0.52, 3.93) |  |
|  |  |  |  |  |
| **NHANES** |  |  |  | 0.56 |
| Female | 1.00 (Ref) | 1.75 (0.19, 9.64) | 4.90 (0.99, 26.9) ^*^ |  |
| Male | 1.00 (Ref) | 0.83 (0.13, 2.97) | 6.84 (2.56, 17.4) ^***^ |  |
|  | **No** | **Yes** |  |  |
| **UKB** |  |  |  | 0.07 |
| Female | 1.00 (Ref) | 1.17 (0.78, 1.75) |  |  |
| Male | 1.00 (Ref) | 1.57 (1.17, 2.10) ^**^ |  |  |

Abbreviations: CHARLS, China Health and Retirement Longitudinal Survey; NHANES, National Health and Nutrition Examination Survey; UKB, UK Biobank.

CHARLS: The model was adjusted for sex, age, marital, education, living standard, BMI, smoking, alcohol drinking, physical activity, hypertension, hyperlipidemia, diabetes, CVD and liver disease including hepatitis B/C infection.

NHANES: The model was adjusted for sex, age, race, marital, education, family income to poverty ratio, BMI, smoking, alcohol drinking, physical activity, HEI and liver diseases including hepatitis B/C infection.

UKB: The model was adjusted for age, sex, ethnicity, BMI, education, TDI, smoking, alcohol drink, physical activity, HEI, hypertension, diabetes, hyperlipidemia, CVD and liver diseases including hepatitis B/C infection.

^*^*P*<0.05; ^**^*P*<0.01; ^***^*P*<0.001.

**Table S4. Association between depression and liver cancer after additionally adjusting for** **antidepressant therapy.**

|  | **Depression** | | |
| --- | --- | --- | --- |
|  | **No** | **Mild** | **Moderate to severe** |
| **CHARLS** | 1.00 (Ref) | 1.04 (0.41, 2.66) | 2.42 (1.08, 5.39) ^*^ |
| **NHANES** | 1.00 (Ref) | 1.26 (0.39, 3.27) | 5.72 (2.43, 13.0) ^***^ |
|  | **No** | **Yes** |  |
| **UKB** | 1.00 (Ref) | 1.38 (1.09, 1.78) ^*^ |  |

Abbreviations: CHARLS, China Health and Retirement Longitudinal Survey; NHANES, National Health and Nutrition Examination Survey; UKB, UK Biobank.

CHARLS: The model was adjusted for sex, age, marital, education, living standard, BMI, smoking, alcohol drinking, physical activity, hypertension, hyperlipidemia, diabetes, CVD, liver disease and antidepressant therapy.

NHANES: The model was adjusted for sex, age, race, marital, education, family income to poverty ratio, BMI, smoking, alcohol drinking, physical activity, HEI, liver diseases and antidepressant therapy.

UKB: The model was adjusted for age, sex, ethnicity, BMI, education, TDI, smoking, alcohol drinking, physical activity, HEI, hypertension, diabetes, hyperlipidemia, CVD, liver diseases and antidepressant therapy.

^*^*P*<0.05; ^**^*P*<0.01; ^***^*P*<0.001.

**Table S5.** **Detailed information on 60 patients.**

| ID | Gender | Age | Tumor size(cm) | MVI | lymphatic node metastasis | Weight(kg)/Height(cm) | AFP(ng/ml) | HbsAg(+/-) | Cirrhosis(+/-) |
| --- | --- | --- | --- | --- | --- | --- | --- | --- | --- |
| 1 | female | 80 | 4*4*3.5 | 0 | 0 | 50/158 | 1.45 | - | + |
| 2 | female | 57 | 4.5*3*3.8 | 0 | 0 | 63 | 2.72 | - | + |
| 3 | male | 45 | 6*6.5*2.5 | 0 | 0 | 65/175 | 3828 | - | + |
| 4 | male | 55 | 1*1*0.4 | 0 | 0 | 70/173 | 2.31 | - | + |
| 5 | male | 40 | 4.5*3*5 | 3 | 0 | 74/175 | 4909 | + | + |
| 6 | male | 55 | 7.5*6*4.5 | 2 | 0 | 83/169 | 81.2 | + | + |
| 7 | female | 71 | 5*4*5 | 1 | 0 | 55/150 | 29960 | + | + |
| 8 | female | 51 | 5*5*3.7 | 1 | 0 | 54/158 | 2205 | + | + |
| 9 | female | 58 | 3*3*2.5 | 1 | 0 | 61/158 | 75.27 | + | + |
| 10 | female | 54 | 8*6.5*5 | 1 | 0 | 48/145 | 11.6 | + | + |
| 11 | male | 61 | 4.5*4*3 | 1 | 0 | 60/167 | 214 | + | + |
| 12 | male | 34 | 4*3*3 | 1 | 1 | 64/65 | 93.18 | + | + |
| 13 | male | 61 | 2.8*2.8*2.5 | 1 | 0 | 60/170 | 1.64 | + | + |
| 14 | male | 66 | 4*2.5*2 | 1 | 0 | 74/165 | 4.32 | + | + |
| 15 | female | 67 | 6*5*4.8 | 0 | 0 | 62/159 | 2.11 | + | + |
| 16 | female | 67 | 9*9*8 | 0 | 0 | 40 | / | + | + |
| 17 | female | 54 | 2.5*2*2 | 0 | 0 | 65/160 | 3.22 | + | + |
| 18 | female | 70 | 6*5*4 | 0 | 0 | 44/151 | 6436.07 | + | + |
| 19 | male | 53 | 5*4.6*4.5 | 0 | 0 | 68/163 | 2.16 | + | + |
| 20 | male | 74 | 6*6.*5 | 0 | 1 | 59/166 | 4.33 | + | + |
| 21 | male | 67 | 10*7*3.5 | 0 | 0 | 61/170 | 323 | + | + |
| 22 | male | 59 | 3*2.5*2 | 0 | 0 | 55/160 | 25.2 | + | + |
| 23 | male | 58 | 8.2*7*6.3 | 0 | 1 | 52/167 | 57.8 | + | + |
| 24 | male | 47 | 3*2.5*1 | 0 | 0 | 65/167 | 6.42 | + | + |
| 25 | male | 76 | 4.2*2.5*2.5 | 0 | 0 | 46/157 | 3.54 | + | + |
| 26 | male | 62 | 4*2.5*2 | 0 | 1 | 63 | 3.01 | + | + |
| 27 | male | 80 | 3*2.6*3 | 0 | 0 | 60/160 | / | + | + |
| 28 | male | 48 | 2*2*1 | 0 | 0 | 62/164 | 93.36 | + | + |
| 29 | male | 60 | 1*1*0.5 | 0 | 0 | 77/172 | 2.63 | + | + |
| 30 | male | 57 | 4.5*4*4 | 0 | 0 | 60/165 | 14.26 | + | + |
| 31 | male | 31 | 2.7*2.3*1.5 | 0 | 0 | 64/175 | 5.18 | + | + |
| 32 | male | 80 | 3*3*2.5 | 0 | 0 | 58/160 | 3.53 | + | + |
| 33 | male | 59 | 3.5*2*2.7 | 0 | 1 | 66/165 | 4.99 | + | + |
| 34 | male | 63 | 4*3*3 | 0 | 1 | 63.5/168 | 200.41 | + | + |
| 35 | male | 40 | 2.5*2.5*2.3 | 0 | 0 | 71/168 | 3.21 | + | + |
| 36 | male | 42 | 3.*2.5*2 | 0 | 0 | 51/158 | 1.01 | + | + |
| 37 | male | 51 | 2.4*1.9*2 | 0 | 0 | 69/172 | 49.49 | + | + |
| 38 | male | 66 | 2.4*1.8*1 | 0 | 0 | 72.5/176 | 3.17 | + | + |
| 39 | male | 40 | 2.2*1.8*1.4 | 0 | 0 | 74.5/184 | 2.04 | + | + |
| 40 | male | 65 | 4.5*4.3*3.8 | 0 | 1 | 60/167 | 4870 | + | + |
| 41 | male | 52 | 7*6.5*5.8 | 0 | 0 | 64/175 | 1.84 | + | + |
| 42 | male | 47 | 4.5*4.5*3.5 | 0 | 1 | 69/173 | 3.08 | + | + |
| 43 | female | 51 | 10*7*4.5 | 1 | 1 | 46/155 | 3.3 | - | - |
| 44 | male | 41 | 2.5*1.5*1 | 1 | 0 | 72/169 | 2.13 | - | - |
| 45 | male | 64 | 9*8*6.5 | 1 | 0 | 60/174 | 1286.68 | - | - |
| 46 | male | 74 | 8*6.5*5 | 1 | 0 | 72/170 | 1.01 | - | - |
| 47 | female | 80 | 11*9.5*7.8 | 0 | 1 | 52/155 | 10720 | - | - |
| 48 | female | 65 | 4.5*3.5*3 | 0 | 0 | 79/162 | 5.34 | - | - |
| 49 | male | 52 | 2.5*2.3*18 | 0 | 0 | 78/175 | 386 | - | - |
| 50 | male | 64 | 6.5*6*5 | 0 | 0 | 70/165 | 2.81 | - | - |
| 51 | male | 64 | 5.6*4*4 | 0 | 0 | 69/168 | 689.4 | - | - |
| 52 | male | 72 | 6*5*4.5 | 0 | 1 | 71/165 | 16.28 | - | - |
| 53 | male | 47 | 7*6*4 | 2 | 1 | 84/185 | 1.08 | + | - |
| 54 | female | 75 | 4.5*4.5*4 | 0 | 0 | 37/142 | 2.5 | + | - |
| 55 | female | 54 | 2.8*2*1.5 | 0 | 0 | 66/165 | 24.2 | + | - |
| 56 | female | 51 | 6*4*4.5 | 0 | 0 | 55/150 | 2.88 | + | - |
| 57 | male | 67 | 5.3*4.6*4.5 | 0 | 1 | 59.5/166 | 1.8 | + | - |
| 58 | male | 44 | 2.3*2*1.5 | 0 | 0 | 65/169 | 11.25 | + | - |
| 59 | male | 44 | 2.6*2.1*2 | 0 | 0 | 77 | 2.22 | + | - |
| 60 | male | 48 | 9.8*8*7 | 0 | 0 | 67/168 | 49.14 | + | - |
| 61 | male | 61 | 7.8*5.5*2.5 | 1 | 0 | 73/147 | 18.84 | + | - |
| 62 | male | 86 | 8.7*6.8*5.5 | 0 | 1 | 87/171 | 3.2 | + | - |
| 63 | male | 67 | 9.0*8.1*4.4 | 0 | 0 | 50/156 | 1.34 | + | - |
| 64 | male | 18 | 9.1*6.2*3.9 | 0 | 0 | 67/152 | 4.14 | + | - |
| 65 | female | 68 | 8.8*8.0*6.9 | 0 | 1 | 89/154 | 6.53 | - | + |
| 66 | female | 85 | 8.5*5.8*4.7 | 0 | 0 | 47/181 | 16582.25 | - | - |
| 67 | male | 24 | 5.1*4.2*1.4 | 1 | 0 | 88/147 | 10.76 | + | + |
| 68 | female | 87 | 8.2*8.1*6.0 | 0 | 0 | 87/157 | 8.02 | + | + |
| 69 | female | 26 | 3.7*3.1*1.0 | 0 | 0 | 89/148 | 18.03 | - | - |
| 70 | female | 29 | 9.4*3.8*1.7 | 0 | 1 | 55/155 | 14.31 | - | - |
| 71 | male | 61 | 4.5*3.8*2.1 | 0 | 1 | 51/152 | 5.8 | + | + |
| 72 | male | 43 | 5.3*4.2*3.2 | 0 | 1 | 56/170 | 11.28 | + | - |
| 73 | female | 67 | 6.9*4.6*2.2 | 2 | 0 | 80/169 | 14.21 | + | - |
| 74 | male | 37 | 4.4*3.6*1.6 | 1 | 1 | 71/153 | 9.84 | + | - |
| 75 | male | 69 | 7.9*4.4*3.2 | 0 | 1 | 68/152 | 2.14 | - | - |
| 76 | female | 42 | 4.6*2.2*2.2 | 0 | 1 | 50/167 | 17.44 | - | - |
| 77 | male | 82 | 6.5*5.4*2.6 | 1 | 1 | 78/155 | 41026 | + | - |
| 78 | female | 74 | 5.6*3.9*1.1 | 0 | 1 | 67/169 | 17.57 | - | - |
| 79 | male | 79 | 4.7*3.1*1.7 | 1 | 1 | 51/175 | 26907.05 | + | - |
| 80 | female | 62 | 9.0*5.1*4.2 | 0 | 1 | 59/166 | 16.08 | + | + |
| 81 | male | 58 | 6.2*2.6*2.2 | 0 | 0 | 71/170 | 9.05 | + | - |
| 82 | female | 38 | 3.3*2.8*1.1 | 0 | 0 | 71/175 | 5.71 | + | - |
| 83 | female | 62 | 6.6*3.4*2.2 | 1 | 0 | 79/165 | 4.73 | + | - |
| 84 | male | 43 | 8.9*7.8*2.6 | 0 | 1 | 69/153 | 0.51 | - | + |
| 85 | female | 57 | 5.1*2.1*1.4 | 2 | 0 | 65/167 | 6.27 | + | - |
| 86 | male | 39 | 5.5*3.6*1.9 | 0 | 1 | 70/175 | 16.12 | + | - |
| 87 | female | 31 | 3.6*3.3*3.2 | 0 | 0 | 60/166 | 11902 | + | + |
| 88 | female | 67 | 6.9*3.8*3.5 | 0 | 0 | 79/165 | 1.09 | - | + |
| 89 | female | 62 | 3.4*2.8*2.1 | 1 | 0 | 88/163 | 8.59 | + | + |
| 90 | male | 26 | 6.2*5.3*4.0 | 0 | 0 | 90/155 | 4.54 | + | - |
| 91 | male | 86 | 9.7*5.1*1.3 | 0 | 0 | 85/150 | 12.78 | + | + |
| 92 | male | 63 | 7.7*6.8*4.3 | 0 | 0 | 71/162 | 4.2 | + | + |
| 93 | male | 88 | 6.3*3.9*3.2 | 1 | 0 | 57/175 | 19.33 | + | + |
| 94 | female | 20 | 3.8*2.9*2.0 | 0 | 1 | 63/165 | 12.03 | - | - |
| 95 | female | 32 | 4.0*3.3*1.1 | 0 | 1 | 69/150 | 40510 | - | - |
| 96 | male | 87 | 8.3*3.7*1.9 | 0 | 0 | 77/176 | 0.78 | + | - |
| 97 | male | 61 | 4.7*2.7*1.2 | 1 | 0 | 62/172 | 23828 | - | - |
| 98 | male | 84 | 5.0*4.1*3.9 | 0 | 1 | 61/152 | 9.05 | + | - |
| 99 | female | 85 | 6.7*3.1*2.7 | 1 | 0 | 53/130 | 9 | + | - |
| 100 | male | 73 | 3.2*2.8*2.1 | 0 | 1 | 53/163 | 11.2 | - | + |

| **Table S6. Antibodies used in this study.** | | | |  |
| --- | --- | --- | --- | --- |
| **Antibody** | **Source** | **Identifier** | **Dilution (Application)** |  |
| Anti- CBX5 Rabbit pAb | Proteintech | Cat#11831-1-AP | 1:1000 (WB) |  |
|  |  |  | 1:500 (IHC) |  |
|  |  |  | 1:300 (IF) |  |
| Anti- PHGDH Rabbit pAb | Proteintech | Cat#14719-1-AP | 1:6000 (WB) |  |
|  |  |  | 1:4000 (IHC) |  |
| Anti- MYC Rabbit pAb | Proteintech | Cat#10828-1-AP | 1:5000 (WB) |  |
|  |  |  | 1:800 (IF) |  |
|  |  |  |  |  |
|  |  |  |  |  |
| Anti-DDDDK Tag (Binds to FLAG tag sequence) Rabbit mAb | Abcam | Cat#ab205606 | 1:10000 (WB) |  |
|  |  |  |  |  |
| Anti-β-actin Mouse mAb | Proteintech | Cat#66009-1-Ig | 1:20000 (WB) |  |
| Anti-AFP Rabbit pAb | Proteintech | Cat#14550-1-AP | 1:500 (IHC) |  |
| Anti-RNF123 Rabbit pAb | Proteintech | Cat#25260-1-AP | 1:2000 (WB) |  |
| Anti-HECW2 Rabbit pAb | Proteintech | Cat#32026-1-AP | 1:1000 (WB) |  |
| Anti-YAP Rabbit pAb | Proteintech | Cat#13584-1-AP | 1:5000 (WB) |  |
| Anti-SOX9 Rabbit pAb | Proteintech | Cat#55152-1-AP | 1:2000 (WB) |  |
| Anti-FXR Rabbit pAb | Proteintech | Cat#25055-1-AP | 1:3000 (WB) |  |
| Anti-CYP7A1 Rabbit pAb | Proteintech | Cat#18054-1-AP | 1:2000 (WB) |  |
| IHC, immunohistochemistry; mAb, monoclonal antibody; pAb, polyclonal antibody; WB, western blot;IF,Immunofluorescence | | | |  |

| **Table S7. Primers for RT-qPCR used in this study.** | |
| --- | --- |
| **Gene Symbol** | **Primer Sequences（5'-3'）** |
| CBX5(Human) | F: ATGGGAAAGAAAACCAAGAG |
|  | R: CAGGACAATCCAAGTTCTTC |
| PHGDH(Human) | F: ATCTCTCACGGGGGTTGTG |
|  | R: AGGCTCGCATCAGTGTCC |
| β-actin(Human) | F:AAGTGTGACGTTGACATCCG |
|  | R:GATCCACATCTGCTGGAAGG |
| Cbx5(Mouse) | F:GACAGGCGCATGGTTAAGG  R:CCTGGGCTTATTGTTTTCACCC |
| Phgdh(Mouse) | F:ATGGCCTTCGCAAATCTGC |
|  | R:AGTTCAGCTATCAGCTCCTCC |
| Cyp7a1(Mouse) | F:TGATCTGGGGGATTGCTGTG |
|  | R: TTCCCCATCAGTTTGCAGGT |
| Cyp8a1(Mouse) | F:AGGCCAGGATGAAGCCAGAC |
|  | R:GGGCGTTGTAGGAGAACTCA |
| Cyp7b1(Mouse) | F:CCCTGCGTGACGAAATTGAC |
|  | R:TCGAACCTAAATTCCCAGGCA |
| Cyp27a1(Mouse) | F: AGCCAAGGCCACGATCCC |
|  | R: ACTAGCCAGATTCACATTGGTGT |
| Abcb11(Mouse) | F: TCCTGGCTCCCTCAAATTCAC |
|  | R: CAGGTAGTGTCAGTGGCCTTT |
| Fxr(Mouse) | F: GATGCTGAAGCTTATGCCGGA |
|  | R: ACTTCTGGGATGGTGGTCCT |
| Sox9(Mouse) | F: GTGCAAGCTGGCAAAGTTGA |
|  | R: TGCTCAGTTCACCGATGTCC |

| **Table S8. The sequences of siRNA and shRNA used in this study.** | | |
| --- | --- | --- |
| **Purpose** | **name** | **Sequence (5'-3')** |
| siRNA(Human) | siCBX5#1 | GAAUUUAUGAAAAAGUAUA |
| siRNA(Human) | siCBX5#2 | GAAGAUUUGAAAUUUUACACU |
| shRNA(Human) | shMYC#1 | CAGTTGAAACACAAACTTGAA |
| shRNA(Human)  sgRNA(Mouse) | shMYC#2  sgPhgdh#1 | CCTGAGACAGATCAGCAACAA  CGAGAACCCTGCCCAGTCAC |
| sgRNA(Mouse) | sgPhgdh#2 | GGGCAGGGCTGGCACAGGTG |

| **Table S9. Primers for ChIP-qPCR used in this study.** | |
| --- | --- |
| **Gene Symbol** | **Primer Sequences（5'-3'）** |
| PHGDH | F: TCATCGCGGGAGGATAAAGC |
|  | R:CCCAGTCATCTCCTCCTCCT |
